# Identification of small molecules affecting the interaction between human hemoglobin and *Staphylococcus aureus* IsdB hemophore

**DOI:** 10.1038/s41598-024-55931-8

**Published:** 2024-04-09

**Authors:** Monica Cozzi, Mariacristina Failla, Eleonora Gianquinto, Sandra Kovachka, Valeria Buoli Comani, Carlotta Compari, Omar De Bei, Roberta Giaccari, Francesco Marchesani, Marialaura Marchetti, Luca Ronda, Barbara Rolando, Massimo Baroni, Gabriele Cruciani, Barbara Campanini, Stefano Bettati, Serena Faggiano, Loretta Lazzarato, Francesca Spyrakis

**Affiliations:** 1 Department of Food and Drug, University of Parma, Parma, Italy; 2 Department of Drug Science and Technology, University of Turin, Turin, Italy; 3 The Herbert Wertheim UF Scripps Institute for Biomedical Innovation & Technology, Jupiter, FL USA; 4 Department of Medicine and Surgery, University of Parma, Parma, Italy; 5 Institute of Biophysics, National Research Council, Pisa, Italy; 6 Molecular Discovery Ltd, Kisnetic Business Centre, Elstree, Borehamwood, Hertfordshire UK; 7 Department of Chemistry, Biology and Biotechnology, University of Perugia, Perugia, Italy

**Keywords:** Biochemistry, Infectious diseases, Bacterial infection, Biophysical chemistry, Drug development

## Abstract

Human hemoglobin (Hb) is the preferred iron source of *Staphylococcus aureus*. This pathogenic bacterium exploits a sophisticated protein machinery called Iron-regulated surface determinant (Isd) system to bind Hb, extract and internalize heme, and finally degrade it to complete iron acquisition. IsdB, the surface exposed Hb receptor, is a proven virulence factor of *S. aureus* and the inhibition of its interaction with Hb can be pursued as a strategy to develop new classes of antimicrobials. To identify small molecules able to disrupt IsdB:Hb protein–protein interactions (PPIs), we carried out a structure-based virtual screening campaign and developed an *ad hoc* immunoassay to screen the retrieved set of commercially available compounds. Saturation-transfer difference (STD) NMR was applied to verify specific interactions of a sub-set of molecules, chosen based on their efficacy in reducing the amount of Hb bound to IsdB. Among molecules for which direct binding was verified, the best hit was submitted to ITC analysis to measure the binding affinity to Hb, which was found to be in the low micromolar range. The results demonstrate the viability of the proposed in silico*/*in vitro experimental pipeline to discover and test IsdB:Hb PPI inhibitors. The identified lead compound will be the starting point for future SAR and molecule optimization campaigns.

## Introduction

Antimicrobial resistance represents one of the main current risks to human health. The emergence of bacteria resistant to last-resort antibiotics is responsible for more than 700,000 annual deaths and, if the trend continues, by 2050, 10 million people worldwide will die each year from antibiotic-resistant infections^1^. High-priority bacteria include the methicillin-resistant (MRSA) and the vancomycin-resistant (VRSA) variants of *Staphylococcus aureus*. *S. aureus* is an extremely elusive Gram-positive coccus that belongs to the so-called ESKAPE group, able to develop resistance against several classes of antibiotics (penicillins, cephalosporins, carbapenems, glycopeptides, and others). In fifteen European countries, more than 10% of bloodstream *S. aureus* infections are caused by MRSA, with a resistance rate close to 50%. To respond to this emerging threat, new targets, new treatments, and alternative therapies are urgently needed.

For all forms of life, iron is a vital nutrient for several cellular processes. In particular, cells in rapid growth such as bacteria require iron for their replication and invasion and, hence, their virulence^2,3^. Considering the importance of iron for bacterial growth, it has been recently proposed to exploit iron starvation for the development of innovative antimicrobials^4,5^, in line with the recently announced WHO “Innovation criteria” that recommend the reinforcement of programs aimed at finding “new targets” and “new mechanisms of action” for antibiotics^6,7^. The host iron is mainly stored bound to proteins, like hemoglobin (Hb), thus reducing its intrinsic reactivity and toxicity with the additional benefit of making the metal unavailable for the growth of pathogens^8,9^. Indeed, host iron sequestration contributes to the so-called nutritional immunity, *i.e.* a form of innate immunity that exploits nutrient limitation to starve bacteria and inhibit their proliferation^10^.

**Figure 1:**
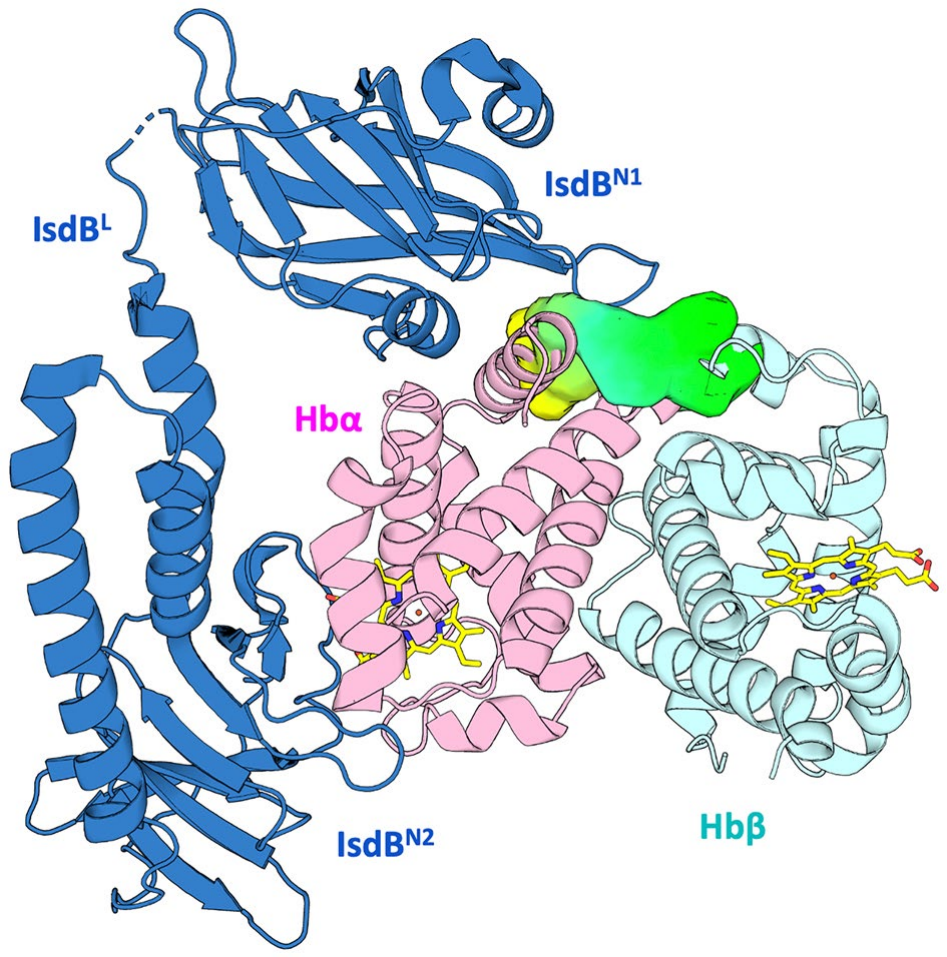
Model of IsdB:Hb complex. The proteins are shown in cartoons (IsdB: blue; Hbα: pink; Hbβ: light blue), the targeted pocket as light green-yellow contour and the heme groups in yellow capped sticks. The structure has been modified from PDB ID 5VMM^30^.

In humans, Hb contains the larger percentage of total body iron (about 60%)^9^, bound to the protein through a heme moiety that has been proven to represent the preferred iron source for some pathogenic bacteria, including *S. aureus*^11^. Indeed, many bacteria can acquire hemic iron from Hb using both soluble and membrane-bound receptors^4^. The Iron-regulated surface determinant (Isd) system of *S. aureus* represents the prototypical system for hemic iron acquisition from Hb in Gram-positive bacteria^12^. It consists of nine proteins (IsdA-I) that allow *S. aureus* to get iron supply from human Hb by binding the host protein, extracting heme and internalizing it to release iron in the cytoplasm^12–14^. Within the Isd system, the Hb receptor IsdB is classified as a virulence factor^15,16^. Deletion of *isdB* gene in *S. aureus* causes a reduction of the pathogen infectivity in animal models^17,18^, and anti-IsdB high-affinity antibodies are expressed in humans as part of innate-like immunity recognizing certain unique structural motifs of infectious pathogens^19^. Moreover, it was recently proved that targeting IsdB and IsdH with a single VHH antibody inhibits *S. aureus* growth^20^. IsdB is anchored on the bacterial cell wall by an LPxTG sortase-recognized sequence and is constituted by two NEAT (NEAr-iron transporter) domains separated by a linker domain (Fig. 1). The two NEAT domains (IsdB^N1^ and IsdB^N2^) share a similar immunoglobulin-like fold, albeit with a very low sequence identity^21^. *S. aureus* IsdB specifically binds human Hb but can extract heme only when iron is in the + 3 oxidation state (methemoglobin, metHb)^22,23^. IsdB^N1^ captures Hb through the Hb-binding motif 163-QFYHYAS-169, while the IsdB^N2^ domain is involved in heme extraction by a heme-binding signature (440-YGDQY-444). At first, IsdB induces Hb tetramer dissociation into dimers. Then, after Hb engagement by IsdB^N1^, IsdB^N2^ extracts the heme following unfolding of Hb F-helix and rotation of the cofactor^22^. Heme extraction by IsdB is completed in about 30 s^24^, with a 2,000-fold rate increase with respect to the non-catalysed reaction^23^. The IsdB:Hb complex is quite stable, with a dissociation constant estimated by SPR spectroscopy of about 70 nM^24^. Both NEAT domains contribute to complex stabilization, which is further increased by the linker domain IsdB^L^^25,26^.

Here we exploited an integrated in silico/in vitro approach to identify, for the first time, non-peptidic ligands able to interfere with the binding of Hb to IsdB, with the far-reaching aim of developing protein–protein interaction (PPI) inhibitors able to interfere with hemic iron acquisition by *S. aureus*^27^. Our approach consists of targeting free Hb, which is released from erythrocytes in the bloodstream by the action of bacterial hemolysins, with small molecules unable to cross the red blood cell membrane, and therefore without interfering with intracellular Hb. To this aim, we screened three different commercial databases, filtered to retain more polar molecules, employing a structure-based virtual screening (SBVS) approach. We decided to target a hollow surface on Hb that is contacted by IsdB^N1^, which is known to be essential for the PPI to occur^17,25^. The 54 selected molecules were assayed for their ability to interfere with IsdB:Hb complex formation by an ad hoc developed immunoassay. The positive hits were further tested by saturation-transfer difference (STD) NMR on Hb to validate their capability of interacting with the target. The affinity to Hb of the most potent molecule was assessed by ITC and found to be in the low micromolar range. This approach demonstrates the possibility of targeting a host protein (Hb) to tackle bacterial metabolism through the inhibition of PPIs, laying the basis for future design, development and optimization of molecules inhibiting the growth of the high priority pathogen *S. aureus*^28,29^.

## Materials and methods

### In silico screening

#### Target preparation and molecular dynamics (MD)

The structures of metHb and metHb complexed with haptoglobin (Hp) were retrieved from the Protein Data Bank (PDB ID 3P5Q and 4X0L, respectively). Residues have been numbered according to PDB ID 3P5Q and 5VMM. Proximal and distal histidines were modelled in the delta-protonated histidine (HID) state, to maintain coordination with the heme iron and the water molecule in the cavity, respectively. Heme parameters were kindly provided by Dr. Leonardo Boechi (University of Buenos Aires), according to Refs.^24,31^. For parameterizing the protein, Amber-ff99SB-ILDN force field, with AmberTools 12, was used^32^. Both Hb structures were submitted to LEaP, to solvate the protein in a 15 × 15 × 15 Å box (buffer distance of 15 Å between the protein and the edge of the box), with a 3-point-charge (TIP3P) model of water; two Na^+^ ions were added to neutralize the system. The obtained coordinates and topology files were converted to .gro and .top with the acpype tool, and submitted to GROMACS 4.6 as input files. The system was minimised in 10,000 steps with the steepest descent algorithm in periodic boundary conditions (PBC). The thermalisation/equilibration stage was divided in four steps: in the first three steps the system was heated up from 0 to 300 K in canonical ensemble (NVT) conditions, the last step was a short MD production of 2 ns in isothermal-isobaric ensemble (NPT) conditions. The production was conducted in NVT, with the temperature fixed at 300 K for 200 ns. The output trajectory was fixed and analysed using GROMACS tools^33^. The clustering was performed with the *medoids* tool (developed by Molecular Discovery Ltd.), able to load multiple structures computationally obtained by MD trajectories, properly centred, and aligned. 20,000 conformations were extracted from each trajectory. An elliptical pocket large enough to cover the targeted region was defined, with delimitations by residues Asn9, Lys16, Glu116 on Hbα and by Asp52 and His116 on Hbβ, in all conformations. For each conformation, the four standard MIFs (H, hydrophobic (DRY), O, N1) were calculated within the pocket space. The resulting molecular interaction field (MIF) values were used as input data for a three-component principal component analysis (PCA), which allowed the trajectory clustering in the score space in which each conformation is associated with a point having three coordinates. Five clusters were generated for each trajectory and redundant centroids in terms of pocket residue conformation were discarded. The remaining conformations, plus the original X-ray structures, were used for the following SBVS analyses.

#### Library preparation

Three libraries of compounds, Vitas, Specs and Enamine, retrieved from the ZINC database^34^ (2,043,809 molecules in total), were screened against the target. For each ligand in the databases, the distribution coefficient at pH 7.4 was calculated using the MoKa software^35^. Molecules with logD < 0 were retained, so that only compounds with a high probability of staying in the bloodstream without crossing cellular membranes were considered. The most probable tautomer and protomer were calculated for each molecule. At the end of the filtering session, more than 100,000 candidates were imported in FLAP and the highest-scored compounds were visually inspected to check the absence of any anomalousness in the molecular structure.

#### SBVS

SBVS experiments were performed with the FLAP suite, developed and licensed by Molecular Discovery Ltd.^36,37^.

The structures of metHb and metHb complexed with Hp, as well as the four selected centroids extracted from the MD trajectories were imported in FLAP. The previously calculated pocket, located at the IsdB^N1^-Hb interface was used. The ligand databases were also generated with FLAP. When building a database, FLAP saves the MIFs associated with each conformation, the FLAP molecular fingerprint, the molecular structure and other information: in this way, the same database can be imported and screened against different targets. During the screening, the MIF quadruplets calculated for each ligand are compared to the quadruplets of the binding site in the target^36^. A score is given for the interaction described not only by every single probe but also by a combination of probes, so that the total set of molecules can be sorted out in decreasing order, choosing the type of interaction. In the context of this work, three probe scores (N1, N1*O, O) were used to rank VS results, for prioritizing molecules with mainly donor (N1 probe ranking), mainly acceptor (O probe ranking) or a combination of donor and acceptor (N1*O probes ranking) character. This choice was made to find polar compounds able to establish anchoring hydrogen bonds with the target, shielding key polar residues on Hb and preventing the recognition by IsdB^N1^. Finally, for each candidate, more than one geometry of the ligand in the binding site was inspected.

#### Molecular docking

GOLD version 5.5 was used to perform docking simulations and the CHEMPLP fitness function was exploited to score the poses^38^. Thr118 was selected as the centroid of a 12 Å cavity. For each molecule, at least ten poses were generated and visually inspected to check the orientation of the complex. Asn9, Lys16, Glu116 on Hbα and Asp52 and His116 on Hbβ were identified as potentially polar anchors for docked ligands. The most promising compounds were chosen according to their docking score as well as to their pose, their capability to establish hydrogen bonds and their chemical diversity.

### In vitro validation of targets

#### Chemicals

All reagents, if not otherwise specified, were purchased from Sigma Aldrich (St. Louis, MO, USA) at the best commercial quality available, and used as received.

#### Protein expression and purification

IsdB was expressed as previously described^24^ and purified by affinity chromatography on a StrepTactin® resin (IBA Lifesciences). Size exclusion chromatography on a Superdex 75 pg HiLoad column (GE Healthcare) connected to an ÄKTA prime (GE Healthcare) was performed to separate the protein from higher molecular weight contaminants and aggregates. The site-directed mutant Y165A (IsdB Y165A) was prepared with standard mutagenesis techniques^39^ and purified to homogeneity by the method optimized for the wild-type (wt) protein.

Human hemoglobin A from non-smoking donors was purified from outdated blood obtained from a blood transfusion centre as described in Ref.^40^.

#### Antibody and immunoassay reagents

Commercial Strep-Tactin®XT coated microplates (IBA Lifesciences) were purchased to exploit the protein C-terminal StrepTag®II for hemophore immobilization on the bottom of the plate. A horseradish peroxidase (HRP)-coupled anti-Hb polyclonal antibody was purchased from Abcam (Ab, Abcam ab19362). A binding buffer (2 mM EDTA, 140 mM NaCl, and 25 mM TRIS/HCl pH 7.6) was used throughout all the assays as suggested by the microplate manufacturer. A washing buffer (binding buffer containing 0.05% Tween-20) was used for all the microplate washing steps. The HRP colorimetric substrate 3,3′,5,5′-tetramethylbenzidine (TMB) was purchased from Acros Organics (Acros Organics, 328051000) and Merck (T4319).

#### Immunoassay optimization

The immunoassay was optimized using the Y165A variant of IsdB. This variant was chosen to limit the risk of oxyHb auto-oxidation. In fact, to increase the sensitivity of the assay for weak Hb binders, Hb concentration in the screening should be kept around its dissociation constant for IsdB. The dissociation constant of Hb for wt IsdB is lower (about 35 nM) than the tetramer/dimer dissociation constant of oxyHb (about 250 nM^24^), so oxyHb in the assays with wt IsdB is expected to be mainly in the dimeric state, which in turn is more prone to auto-oxidation^41^. On the other hand, the dissociation constant of oxyHb for the IsdB Y165A variant is expected to be significantly higher based on literature data^17^ and was indeed measured to be of ~ 80 µM in this work. This implies that the concentration of Hb required to half-saturate IsdB is high enough to stabilize the tetrameric form of Hb, less prone to auto-oxidation.

Strep-Tactin®XT-coated microplate wells were incubated O/N at 4 °C with 2 pmol StrepTag®II- Y165A IsdB, following producer’s recommendations. The plate was washed with washing buffer and 30 µM oxyHb was added and incubated for 1 h at 4 °C. After washing the plate, Ab was added and incubated for 1 h at 4 °C. After washing, 100 μL of HRP substrate was added to the wells and the reaction developed for 30 s at 4 °C before stopping it with 100 μL of 1 N H_2_SO_4_. Different Ab dilutions (between 1:100 and 1:1500) were used to assess the effect of Ab concentration on the assay output under these conditions (Fig. S1A). While a dose-dependence of the measured signal was apparent, further dilution of the Ab (between 1:1000 and 1:10,000) did not affect the measured affinity (Fig. S1B). Using 2 pmol StrepTag®II- Y165A IsdB, 30 µM oxyHb, 1:1000 Ab dilution and three washings at each step, the effect of Hb incubation time with IsdB-functionalized wells was assessed. The signal intensity measured after 1 h did not change after 2- and 3-h incubation (Fig. S1C). The effect of increasing the number of washings from three to six on the amount of bound Hb detected was also investigated and confirmed to be negligible (Fig. S1D). The optimized protocol is reported hereafter.

Plate functionalization: 2 pmol StrepTag®II-IsdB incubated O/N at 4 °C in the Strep-Tactin®XT coated microplate. The solution is removed, and the wells washed three times with washing buffer.

Hb binding: oxyHb solution in binding buffer is added to the plate and incubated for 1 h at 4 °C. The solution is removed, and the plate washed three times with washing buffer.

Detection: an Ab solution diluted 1:1000 in binding buffer is added to the well and incubated for 1 h. The solution is removed, the plate is washed three times with washing buffer and 100 μL of HRP substrate solution is added and allowed to incubate for 30 s at 4 °C. The reaction is stopped by the addition of 100 μL 1 N H_2_SO_4_ solution. Absorption of HRP reaction product is recorded at 450 nm with a microplate reader (Dynamica Halo LED 96), and background values from TMB solutions are subtracted from each reading.

The dependence of A_450_ on the concentration of Hb was fitted to an isothermal equation (Eq. (1)) to calculate the dissociation constant for the complex.

1 $${A}_{450}=\frac{{A}_{max}\cdot [Hb]}{{K}_{D}+[Hb]}.$$

#### Screening of potential inhibitors of IsdB-Hb complex formation

Commercial and synthesized compounds were tested at 1 mM concentration. Since investigated molecules were expected to bind Hb, the solutions containing Hb (at a sub-saturating concentration of 30 µM) and each potential inhibitor (110 µL/well) were pre-incubated for one hour at 4 °C in the dark in a not-functionalized plate (Sarstedt AG & Co., 83.3924.500). The Hb-compound solution was then added to the functionalized well and the immunoassay procedure was followed.

The results are expressed as percentage of bound Hb, calculated with respect to the absorbance at 450 nm of the negative control. Each measurement is the average of at least three replicates, except for C35 commercial compound (two replicates). This compound gave irreproducible results in the first screening, with one outlier in the triplicate. Since the inhibitory activity of C35, despite being highly variable, appeared promising, a higher number of replicates was performed following the initial screening. The resulting average percent of bound Hb after 12 experiments was 0.42 ± 0.27. This compound was later demonstrated to rearrange due to dissolution in an alkaline aqueous solution.

#### Compound solubilisation

The molecules under investigation were purchased from commercial suppliers (Enamine Ltd., Specs, and Vitas-M Laboratory, Ltd.) or synthesized in-house. The chemical structures of the compounds together with their MW are reported in Table 1. The first batch (C01-30) was purchased from Specs and Vitas-M Laboratory Ltd., while the second batch (C31-54) was purchased from Enamine Ltd. Deuterated (D_6_)-dimethyl sulfoxide (DMSO) used to solubilize the compounds was purchased from VWR Chemicals.

**Table 1:** Properties of the commercial molecules identified by virtual screening and tested in vitro. Compou﻿nds C01-C30 w﻿ere purchased from Specs and Vitas-M Laboratory Ltd., while compounds C31-C54 were purchased from Enamine Ltd.

ID	Vendor ID	Structure	MW (Da)
C01	SPECS: AE-848/30697010		212.3
C02	SPECS: AN-524/12525005		338.4
C03	SPECS: AJ-333/36116036		286.4
C04	SPECS: AJ-264/34032018		246.3
C05	SPECS: AO-365/15404040		236.3
C06	SPECS: AL-466/21162036		441.4
C07	SPECS: AG-205/03647037		277.3
C08	SPECS: AN-329/40675062		426.5
C09	SPECS: AJ-292/41694677		344.4
C10	SPECS: AN-465/42889348		315.8
C11	SPECS: AN-465/42889125		281.4
C12	SPECS: AP-263/41946485		478.6
C13	SPECS: AN-698/42116797		487.9
C14	SPECS: AN-329/42607819		357.4
C15	SPECS: AN-465/43421792		268.4
C16	SPECS: AS-871/43475868		244.3
C17	VITAS: STK326375		294.3
C18	VITAS: STK797000		517.6
C19	VITAS: STL286070		303.5
C20	VITAS: STK649708		402.5
C21	VITAS: STK650618		479.5
C22	VITAS: STK660860		479.5
C23	VITAS: STK648935		390.4
C24	VITAS: STK648766		463.5
C25	VITAS: STK651328		470.5
C26	VITAS: STK377176		331.3
C27	VITAS: STK370870		273.3
C28	VITAS: STK368826		648.8
C29	VITAS: STK147714		344.4
C30	VITAS: STL283606		402.9
C31	ENAMINE: Z135426094		373.4
C32	ENAMINE: Z54759841		334.4
C33	ENAMINE: Z595841084		330.4
C34	ENAMINE: Z595834718		281.3
C35	ENAMINE: Z54346381		394.4
C36	ENAMINE: Z3046636218		302.4
C37	ENAMINE: Z367450928		355.4
C38	ENAMINE: Z929732930		305.3
C39	ENAMINE: Z1455661524		323.4
C40	ENAMINE: Z18356574		381.5
C41	ENAMINE: Z2385165184		301.4
C42	ENAMINE: Z1757374096		301.4
C43	ENAMINE: Z1443877863		340.4
C44	ENAMINE: Z991585878		261.3
C45	ENAMINE: Z645334138		350.4
C46	ENAMINE: Z990000736		222.3
C47	ENAMINE: Z989410844		302.4
C48	ENAMINE: Z1757876625		339.4
C49	ENAMINE: Z1931004203		384.4
C50	ENAMINE: Z1139246176		341.4
C51	ENAMINE: Z1127202154		342.4
C52	ENAMINE: Z1139642738		323.4
C53	ENAMINE: Z1259041010		306.3
C54	ENAMINE: Z18242550		263.3

The commercial compounds were solubilized in aqueous solutions to reach a 50 mM concentration, taking advantage of the pH and concentration-solubility profile estimated by Chemicalize (https://chemicalize.com/ developed by ChemAxon). When the molecules resulted partially or completely insoluble, the pH was adjusted based on their estimated pH-solubility profiles using either HCl or NaOH concentrated stocks. A cycle of sonication with an ultrasonic bath (Bormac CE-5700, 41300303) was performed for those compounds that resulted insoluble after pH adjustment. If sonication was insufficient, compounds were also heated in a thermoblock at 60 °C for ten minutes. For the compounds that were still not soluble, D_6_-DMSO was added. For some compounds, complete solubilization required to decrease the concentration of the stock solution. In Table 2 are reported the compounds that required dilution and/or addition of DMSO for solubilization.

**Table 2:** DMSO addition for compound solubilization.

Compound ID	Stock H_2_O (mM)
C02	19
C17	25 (50% D_6_-DMSO final concentration)
C18	25 (50% D_6_-DMSO final concentration)
C28	25 (50% D_6_-DMSO final concentration)
C33	50 (20% D_6_-DMSO final concentration)
C34	33 (50% D_6_-DMSO final concentration)
C40	50 (20% D_6_-DMSO final concentration)
C44	44 (50% D_6_-DMSO final concentration)
C54	50 (20% D_6_-DMSO final concentration)

The in-house synthesized compounds were solubilized at 100 mM concentration in D_6_-DMSO. We indicated with an asterisk (*) the in-house synthesized compounds to distinguish them from the commercial ones. C35* dissolved in D_6_-DMSO resulted stable by NMR and liquid chromatography, differently from commercial C35 dissolved in alkaline aqueous solution, which underwent a rearrangement due to the basic environment (data not shown). Molecules that resulted partially or completely insoluble were subjected to the same procedure followed for the commercial molecules, except for pH adjustments. The compounds that were highly insoluble were produced as TRIS salts. The chemical structures of the synthesized compound derivatives together with their MW are reported in Table 1.

Absorption spectra of the commercial compounds alone or in the presence of oxyHb were recorded with a microplate reader (TECAN Spark® 10 M) in a 384-well, UV-transparent microplate (Greiner UV-STAR® plate—781801). The compounds were diluted in binding buffer to a 2 mM concentration, and the pH of the solutions was adjusted in the range 6.5–7.5. Compounds were then tested at 1 mM concentration to assess for any absorbance around 450 nm that might interfere with the immunoassay. The effect of compounds on the oxyHb spectrum was assessed under the same conditions in the presence of 10 µM oxyHb. The spectrum was acquired after 2 h, a delay comparable to the whole duration of the assay, including the preliminary incubation of the compound with Hb. All the incubation steps were performed at 4 °C, as in the immunoassay. Spectra were subtracted from the contribution of the blank and the compound.

### Compounds synthesis

#### Chemicals

All chemicals were purchased by Sigma-Aldrich and used as received. Anhydrous sodium sulfate (Na_2_SO_4_) was used as drying agent for the organic phases. Organic solvents were removed under reduced pressure at 30 °C. Synthetic-purity solvents were used.

#### Instrumentation

Reactions were monitored by thin-layer chromatography on silica gel plates (60F-254, E. Merck) and visualized with UV light. ^1^H and ^13^C NMR spectra were recorded on a Jeol ECZ-R 600, at 600 and 150 MHz, respectively, using SiMe_4_ as the internal standard. The following abbreviations are used to designate peak multiplicity: s = singlet, d = doublet, t = triplet, q = quartet, quint = quintet, m = multiplet, bs = broad singlet. ESI–MS spectra were recorded on a Micromass Quattro API micro (Waters Corporation, Milford, MA, USA) mass spectrometer. Data was processed using a MassLynx System (Waters). The reverse-phase HPLC analyses that allowed the determination of purity for all compounds were performed with an HP 1100 chromatograph system (Agilent Technologies, Palo Alto, CA, USA) equipped with a quaternary pump (model G1311A), a membrane degasser (G1379A), a diode-array detector (DAD) (model G1315B) integrated into the HP1100 system. Data analysis was done using an HP ChemStation system (Agilent Technologies). The analytical column was a LiChrospher RP-18e 100 A (250 × 4.6 mm, 5 μm particle size) (Merck, Darmstadt, Germany). The mobile phase consisted of acetonitrile/water (80/20 v/v) with 0.1% trifluoroacetic acid and the flow-rate was 1.2 mL/min. The injection volume was 20 μL (Rheodyne, Cotati, CA). All compounds were ≥ 95% pure.

**Scheme 1:**
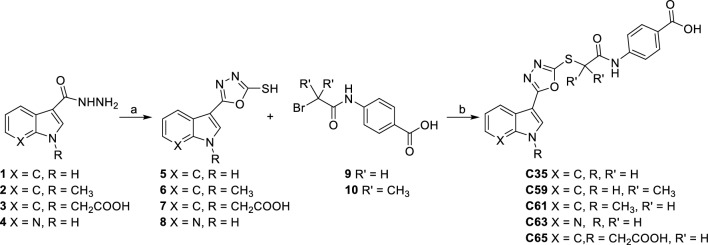
Synthesis of C35, C59, C61, C63 and C65. (**a**) KOH, CS_2_, EtOH, reflux, 4 h. (**b**) K_2_CO_3_, acetone or CH_3_CN, reflux.

Hydrazide **1** was obtained by reaction of commercially available methyl 1H-indole-3-carboxylate with hydrazine in EtOH under reflux. Reaction of hydrazide in the presence of KOH and CS_2_ yielded the oxadiazole-thiol derivatives (**5**–**8**). Nucleophilic substitution between oxadiazole-thiol derivatives and bromoacetamido-benzoic acid derivatives (**9**, **10**) in acetone or acetonitrile under reflux, in presence of K_2_CO_3_, gave **C35**, **C59**, **C61**, **C63** and **C65** (Scheme 1).

**C35**, **C59**, **C61**, **C63** and **C65** were then salified with TRIS to increase their solubility in water (**C35 TRIS**, **C59 TRIS**, **C61 TRIS**, **C63 TRIS** and **C65 TRIS**).

**Scheme 2:**
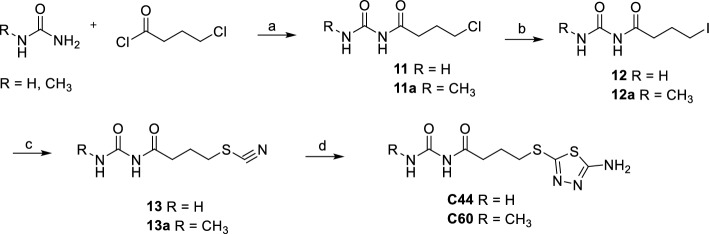
Synthesis of C44 and C60. (**a**) H_2_SO_4_, benzene, reflux for 3 h then r.t. for 12 h, 80%, 84%. (**b**) NaI, acetone, reflux, 8 h, 62%, 79%. (**c**) KSCN, acetone, reflux, 4 h, 65%, 90%. (**d**) Thiosemicarbazide, TFA, reflux, 4 h, 29%, 30%.

Urea or 1-methylurea were reacted with 4-chlorobutanoyl chloride in benzene and H_2_SO_4_, under reflux for 3 h and then at r.t to obtain **11** or **11a** chlorides. **11** and **11a** were converted into their respective iodinated derivatives **12** and **12a** by reaction with NaI in acetone under reflux. The thiocyanates derivatives **13** and **13a** were obtained by reaction of **12** and **12a** with KSCN in acetone under reflux. The reaction of **13** and **13a** with thiosemicarbazide in TFA under reflux gave the final compounds **C44** and its methylated derivative **C60** (Scheme 2).

**Scheme 3:**
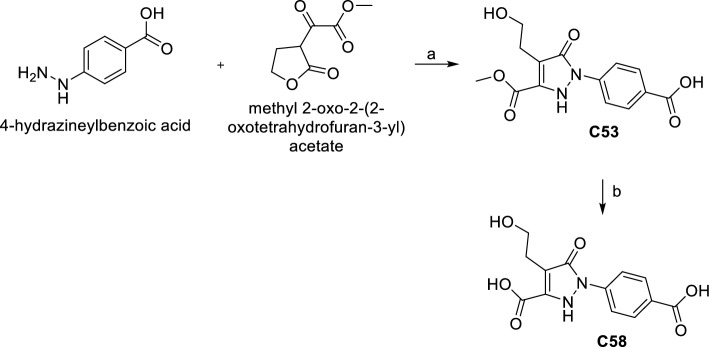
Synthesis of C53 and C58. (**a**) Et_3_N, CH_3_COOH, MeOH, r.t. 24 h, 37%. (**b**) 1 M NaOH, MeOH, r.t. 12 h, 88%.

**C53** was obtained by a reaction between 4-hydrazineylbenzoic acid and methyl 2-oxo-2-(2-oxotetrahydrofuran-3-yl) acetate in MeOH/CH_3_COOH in the presence of Et_3_N at r.t. The hydrolysis of **C53** with 1 M NaOH in MeOH gave the acid derivative **C58** (Scheme 3).

The experimental protocol and characterization for the synthetized compounds are reported in the Supplementary Information.

### Validation of the binding of the most promising compounds to Hb

#### STD-NMR

Hb samples for STD-NMR were diluted at a concentration of 20 µM (heme concentration) in a 50 mM sodium phosphate buffer, pH 7.6, prepared with 100% D_2_O, previously equilibrated with CO. Commercial compounds C35, C41, C44, and C53 and in-house synthesized compounds C35* and C53* were analysed. The concentrated stocks of the commercial compounds were prepared in water, as previously reported, while the in-house synthesized compounds were dissolved in D_6_-DMSO before further dilution in the measuring buffer. Compounds were added to the protein solution to a final concentration of 1 mM (molar ratio of Hb/compound of 1:100).

STD-NMR experiments were performed using a Jeol ECZ-R 600 (600 MHz spectrometer) at 25 °C. NMR spectra were acquired with 64 k data points and 256 scans using a spectral window of 9025 Hz. Water signal suppression was carried out to eliminate signals from trace amounts of H_2_O using a watergate pulse sequence. For selective saturation, cascades of Gaussian-shaped pulses (field strength of 92 Hz) with a length of 30 ms were applied. An interpulse delay of 3 ms between successive pulses was applied, for a saturation time of 6 s. On-resonance and off-resonance spectra were obtained by selective irradiation at 0.6 ppm and 40 ppm, respectively. A Tp1 filter of 50 ms was applied to remove protein peaks. Spectra were analysed using MESTRENOVA 14.2.1 (Mestrelab Research, S.L, Santiago de Compostela, Spain). Group Epitope Mapping (GEM) was performed on the STD spectra assigning the highest saturated signal to 100%, and all other peaks were normalized accordingly.

#### Isothermal titration calorimetry (ITC)

Experiments were performed in triplicate at 25 °C using 100 µM HbCO (heme concentration, hence 25 µM Hb tetramer, the species present in solution at this concentration) and 500 µM C35*. The compound used was a C35* stock without TRIS (i.e. not a TRIS salt), since TRIS is notoriously not ideal as buffer for ITC given its high ionization enthalpic contribution^42^. Indeed, the first attempts with C35* TRIS salt did not give satisfactory results (data not shown). All solutions were prepared in a 50 mM HEPES buffer, pH 7.6. C35* was first dissolved in a concentrated 100 mM stock in DMSO and then diluted in the final buffer to a concentration of 500 µM, at which the compound was soluble. Solutions were degassed for 10 min under vacuum before the titration. HbCO was obtained by adding to the oxyHb stock a buffer previously equilibrated with CO. DMSO was added to the Hb solution at a final concentration of 0.5% to balance the amount of DMSO in the C35* solution.

ITC titrations were carried out using a MicroCal PEAQ-ITC (Malvern, Malvern, UK). C35* was added to the instrument measurement cell, containing 200 μL of HbCO, by a first addition of 0.4 μL and 18 subsequent additions of 2 μL. A time interval of 150 s was set between the addition of each aliquot of C35*. To subtract the dilution heat, an experiment (also in triplicate) was performed in which the reaction cell was filled only with the buffer solution with 0.5% DMSO, while the syringe was filled with 500 µM C35*. Experiments were performed under continuous stirring at 750 rpm. A UV–vis spectrum of the protein was acquired after the titration and confirmed that HbCO did not oxidize during the experiment (data not shown).

Binding isotherms were obtained by the integration of each injection peak followed by subtraction of the control (dilution) experiment. The Hb concentration used for the fitting was 25 µM (concentration of the tetramer). Titration curves were fitted using a model containing one set of identical sites using the MicroCal PEAQ Analysis Software version 1.40 developed by Malvern (Malvern Instruments Ltd.: Microcal PEAQ-ITC Analysis Software, Malvern, UK).

## Results and discussion

### Compound selection

In order to interfere with the IsdB:Hb interaction, we choose to target Hb instead of IsdB for two main reasons. First, targeting the hemophore would apply selective pressure on *S. aureus*, likely leading to novel resistant strains. Second, occupying the Hb region recognized by IsdB (and, possibly, other receptors as IsdH) would offer the advantage of interfering with NEAT-containing hemophores produced by other pathogens, and not only by *S. aureus*^43,44^.

**Figure 2:**
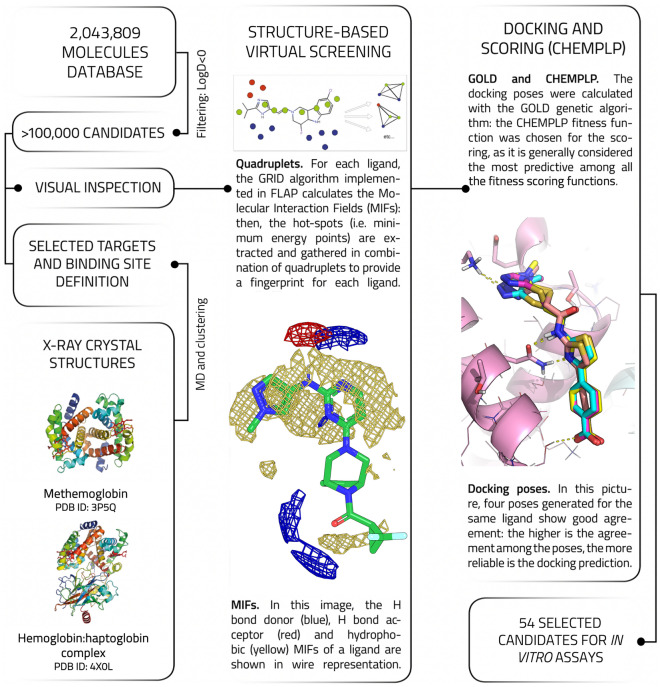
Workflow of the compound selection process.

The crystal structure of the IsdB:Hb complex^30^ solved at 3.6 Å (PDB ID 5VMM) was taken as a model to understand which contacts occur at the interface between the two proteins. In the asymmetric unit of the crystal, the hemophore IsdB binds the α chain of an αβ Hb dimer (Fig. 1). The selected structure shows a dense network of contacts between Hb and all three IsdB domains (IsdB^N1^, IsdB^L^ and IsdB^N2^). A detailed description of the interactions and of the residues involved in them on both Hb and IsdB sides is reported in Ref.^24^. The analysis of isolated IsdB domains has reported that IsdB^N1^ is involved in the recognition and first interaction with Hb^26^. IsdB^L^ contributes to the initial binding and places IsdB^N2^ in front of the heme cofactor bound to Hb. Finally, IsdB^N2^ pulls out the heme and stabilizes it through a new coordination bond^45^. Within the druggability assessment, the heme-containing cavity in Hb was initially considered, but its highly hydrophobic nature and the flattened shape made it unsuitable. Instead, an upper groove of Hb characterized by hydrophobic and polar interactions with IsdB^N1^ was found. Our hypothesis argues that disrupting these contacts could affect the formation of the IsdB:Hb complex, thus compromising heme transfer and iron supply. The Hb groove recognized by IsdB^N1^ was thus chosen as the binding site for virtual screening analysis (Fig. 2 and Fig. S2). As expected, the same groove is visible at the IsdH^N2^:Hb interface. Residues forming the Isd loop facing the pocket have limited similarity: His241-Phe242-Asn243-Asn244-Lys245 on IsdB^N1^ correspond to Glu442-Tyr443-Gly444-Glu445-Asn446 on IsdH^N2^ (Fig. S2).

Given the poor resolution of the 5VMM model, and the distorted Hb structure (unfolded F-helix), Hb conformations used for the following simulations were retrieved from two different crystallographic structures: metHb^46^ and Hb bound to haptoglobin ^47^. The last structure was used since it is well known that Hp plays an essential role in iron homeostasis by scavenging free Hb in the bloodstream, avoiding oxidative stress and limiting bacterial access to the essential micronutrients^48,49^. Targeting the selected Hb groove is likely to interfere with the IsdB^N1^-Hb interaction without affecting Hb recognition by Hp^9^, as shown in Fig. S3.

Considering that the Hb tetramer needs to dissociate into αβ dimers to allow heme extraction by IsdB^22,^ all simulations were performed on Hb in the dimeric state (with or without Hp).

As a preparatory step for the SBVS procedure (for the general pipeline see Fig. 2), we performed 200 ns-long plain MD simulations on both dimeric metHb and Hb:Hp complex to take into account slightly different conformations of Hb residues side-chains due to protein intrinsic dynamics. It has been extensively demonstrated, by us and others, that including target flexibility drastically improves the performance of VS campaigns^37,50,51^. Both metHb and Hb:Hp trajectories were clustered with the *medoids* tool, developed by Molecular Discovery Ltd., according to the diversity of the pocket molecular interactions fields. Five reference conformations (medoids) were obtained for both metHb and Hb-Hp complex. Six out of ten medoids were redundant in terms of pocket side chain conformations, and were thus discarded. The remaining four medoids and the two original X-ray structures were used as templates for the SBVS campaign, that was carried out with the software FLAP (Molecular Discovery Ltd.)^36,37,52^.

Compounds to be screened were retrieved from three different commercial libraries (Specs, Vitas and Enamine) for a total amount of 2,043,809 molecules. The library was then filtered for logD < 0 to retain more hydrophilic compounds. Indeed, the ultimate goal is to identify molecules that could target free Hb into the bloodstream without penetrating cell membranes and affecting intracellular Hb. A total number of more than 100,000 compounds was retained and submitted to SBVS. Screened ligands were sorted out using FLAP N1, O, and N1*O scores, to prioritize compounds with hydrogen bond donor/acceptor groups capable of interacting with the polar residues lining the pocket of the binding site (Asn9, Lys16, Glu116, Thr118, and Lys 125 on Hbα, Arg30 and Asp52 on Hbβ). The VS campaign led to the selection of about 500 molecules, which were forwarded to a more accurate molecular docking analysis with GOLD^38^. Indeed, molecular docking was aimed at further filtering out compounds selected by the VS campaign, giving insight into the ligand binding mode in the target pocket. The most promising compounds were chosen according to their docking score as well as to their pose, their capability to establish hydrogen bonds and their chemical diversity. Generally, ligands showing conserved poses and contacts were prioritized. At the end of the VS and docking campaign (Fig. 2), 54 molecules were selected to be tested in vitro for their capability of interfering with IsdB:Hb complex formation (Table 2).

**Figure 3:**
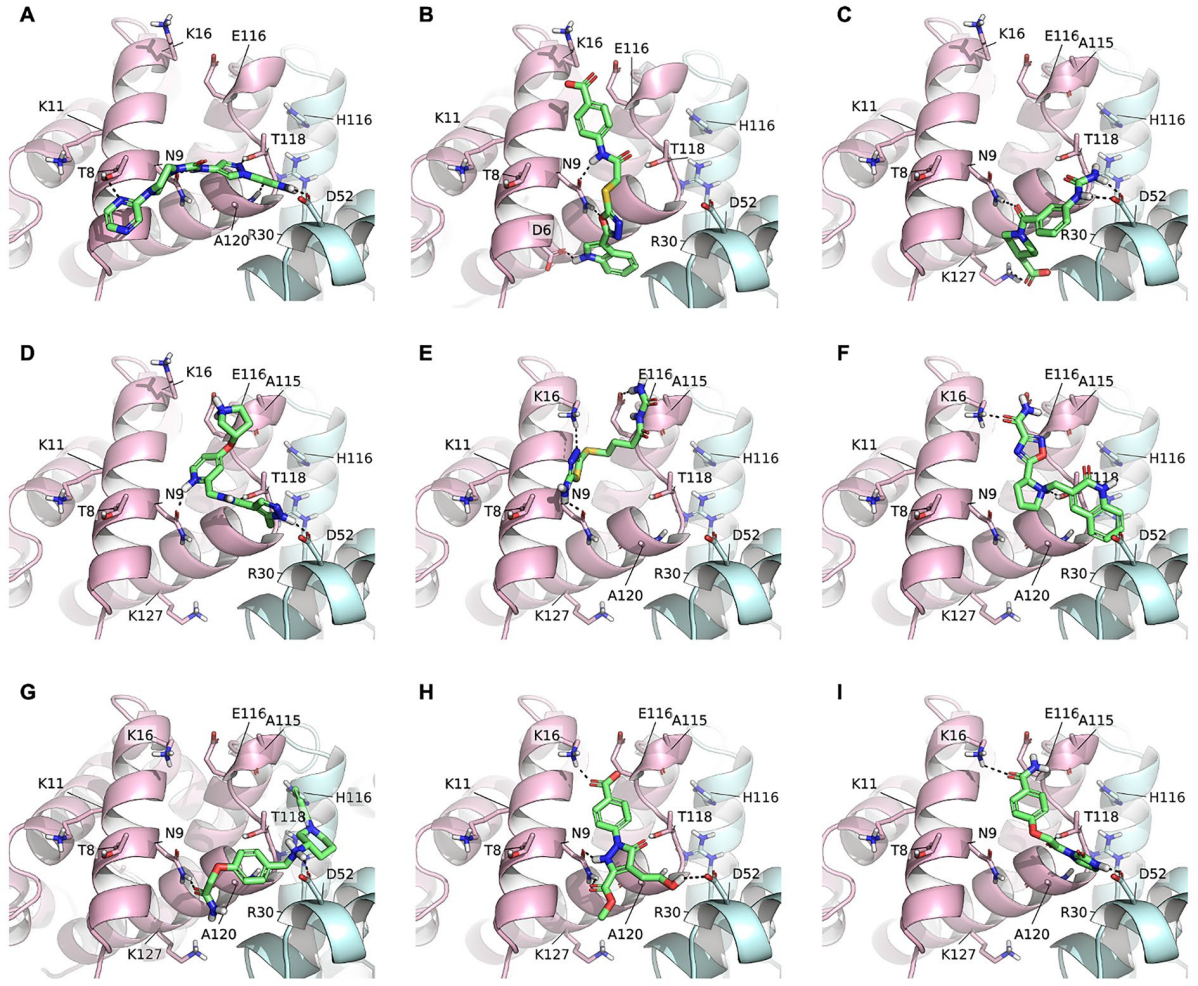
Example of docking poses of selected compounds. (**A**) C33; (**B**) C35; (**C**) C38; (**D**) C41; (**E**) C44; (**F**) C48; (**G**) C50; (**H**) C53; (**I**) C54. The protein is shown in cartoon (Hbα: pink; Hbβ: light blue; PDB ID 3P5Q), the ligands in green capped sticks, hydrogen bonds are shown as black dashed lines.

As an example, some promising compounds are shown in Fig. 3. Compound 33 (Fig. 3A) is apparently able to bridge the two Hb chains assuming an elongated conformation, interacting with Thr8, Asn9, Thr118 and Ala120 on chain α, and forming a salt bridge with Asp52 on chain β. Compound 35 (Fig. 3B) vertically inserts in the Hb chain α, contacting Asp6, Asn9 through a double interaction, and Lys16 through an electrostatic contact. Compound 38 (Fig. 3C) slightly resembles compound 33, bridging Asn9α to Asp52β, but is also able to extend up to Lys127α. Compound 41 (Fig. 3D) contacts both Hb chains by means of H-bonds formed with Asp52β, Asn9α and a salt bridge with Glu116α. Compound 44 (Fig. 3E), a little shorter than 41, only contacts Hbα residues: Asn9, Lys16 and Glu116. Compound 48 (Fig. 3F) again reaches Hbβ through a cation-π contact made by the aromatic ring with Arg30, while forming H-bonds to Lys16, Glu116 and Thr118 on Hbα. Similarly, Compound 50 (Fig. 3G) assumes a position like that of compound 38, contacting Asn9 on Hbα and Asp52 on Hbβ. An additional π–π interaction is likely formed between the pyridazine ring and His116 on Hbβ. Compounds 53 and 54 (Fig. 3H,I) interact with both Hb chains, reaching up to Asp52β. As expected, poor hydrophobic contacts are made in general by all compounds, considering the low logD and the external localization of the pocket, mainly lined by polar or charged residues. Similar poses and interactions to those shown in Fig. 3 are maintained by the other selected compounds (Table 1). It has however to be stated that the suggested poses only correspond to an estimation of possible binding modes.

To verify our predictions, the 54 selected compounds were purchased and submitted to the following in vitro analyses to evaluate their capability of interfering with IsdB:Hb complex formation.

### In vitro evaluation of potential inhibitors

#### Development of an immunoassay for the detection of IsdB:Hb complex formation

**Figure 4:**
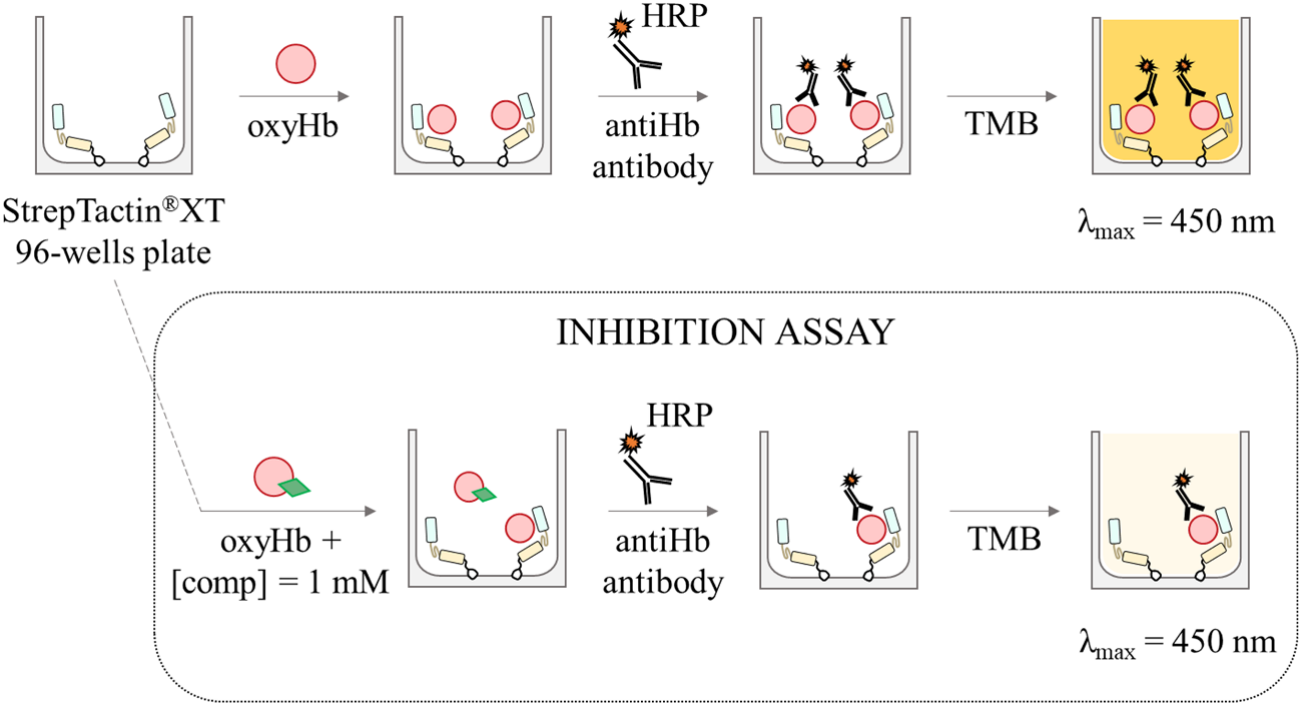
General scheme of the immunoassay. IsdB is immobilized on the bottom of a StrepTactin®XT 96-well plate exploiting its C-terminal StrepTag®II. Hb, alone (top scheme) or after pre-incubation with the compounds at 1 mM concentration (bottom scheme), is then added to the wells. After washing steps, the antiHb HRP-conjugated antibody is added, followed by the substrate 3,3′,5,5′-tetramethylbenzidine (TMB). The reaction is blocked by H_2_SO_4_ and the signal intensity is measured at 450 nm.

We decided to develop a platform for the screening of compounds based on their ability to interfere with IsdB:Hb complex formation. The general scheme for the assay is shown in Fig. 4.

**Figure 5:**
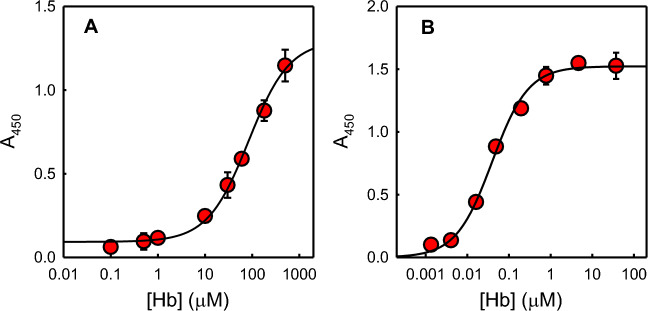
Hb binding to IsdB-functionalized plates followed by immunodetection. (**A**) Titration of Y165A IsdB with Hb. 2 pmol StrepTag®II-Y165A IsdB were used to functionalize a Strep-Tactin® XT-coated microplate. Increasing concentrations of Hb in binding buffer were added to distinct wells of the plate and incubated at 4 °C. A 1:1,000 diluted Ab was added to the wells and incubated at 4 °C for 1 h in the dark. Detection was carried out with the TMB substrate incubated for 30 s at 4 °C. The data points represent the average of three replicates. The line through the datapoints is the fitting to Eq. (1) with a K_D_ = 84 ± 11 μM. (**B**) Titration of wt IsdB with Hb. 2 pmol StrepTag®II-IsdB were used to functionalize a Strep-Tactin® XT-coated microplate. Increasing concentrations of Hb in binding buffer were added to distinct wells of the plate and incubated at 4 °C. A 1:1000 diluted Ab was added to the wells and incubated at 4 °C for 1 h in the dark. Detection was carried out with the TMB substrate incubated for 30 min at room temperature. The data points represent the average of three replicates. The line through the datapoints is the fitting to Eq. (1) with a K_D_ = 39 ± 4 nM.

Briefly, Hb is added to an IsdB-functionalized StrepTactin® 96-well plate, and the amount of Hb bound to IsdB is measured by adding a polyclonal anti-Hb antibody conjugated to horseradish peroxidase (HRP), followed by addition of the chromogenic substrate that is converted into a product maximally absorbing at 450 nm. OxyHb was used in the assay because heme transfer does not take place from this species^22,24^, and the detection of IsdB:Hb complex is not complicated by the following Hb dimerization and heme extraction leading to dissociation from the hemophore^22^. The assay was optimized using a low-affinity variant of IsdB, Y165A IsdB^17^. Indeed, to increase the sensitivity of the screening, Hb concentration should be kept around its dissociation constant for IsdB, i.e. about 35 nM for wt IsdB, a value significantly lower than the tetramer/dimer dissociation constant of oxyHb, about 250 nM^24^. Under these conditions, Hb is mainly in the dimeric form, that is more prone to autoxidation to metHb, especially in the presence of IsdB^22^. MetHb, in turn, is a good substrate for heme extraction by IsdB and the resulting apoHb might dissociate from IsdB^24^. Using Y165A IsdB, a higher concentration of Hb could be used for the assay, stabilizing the tetrameric form that is less prone to autoxidation. Notably, the binding pocket identified for SBVS is distant from Y165, thus the Y165A substitution is not expected to affect the binding of potential inhibitors (Fig. S2). The optimization of the assay was carried out as described in Materials and Methods (Fig. S1). The assay was used to quantitatively estimate the binding of Hb to Y165A IsdB, by a dose–response curve (Fig. 5A). The dissociation constant for the Y165A IsdB:Hb complex was estimated to be 80 ± 11 μM, in agreement with previously published functional data that demonstrated for this variant a reduced ability to acquire heme from Hb and a reduced virulence^17^. As a control, the affinity of Hb for wt IsdB was assessed using the same set-up used for Y165A IsdB (Fig. 5B). The dissociation constant was 39 ± 4 nM, in very good agreement with published SPR data^24^.Finally, we tested if DMSO from the stock solutions of those compounds which were dissolved in 20–50% DMSO (commercial compounds) or 100% DMSO (in-house synthesized) could affect the assay. The presence of 2% DMSO (the maximum concentration in the assay buffer after addition of the compounds at a final concentration of 1 mM) did not affect complex formation (97 ± 9% of bound oxyHb compared to the control experiment (100 ± 4%)).

### Identification of ligands affecting IsdB:Hb interaction

The 54 compounds selected by VS were tested in vitro using the in-house developed immunoassay. The compounds were diluted to 1 mM in binding buffer and the pH of the solution was measured to check that it was maintained at 7.6 ± 0.5.

Absorption spectra of the compounds in solution were collected between 350 and 700 nm to exclude any interference with the determination of Hb oxidation (vide infra) and with the absorbance measurement at 450 nm of the immunoassay (Fig. S4). The absorption spectra of Hb solutions were collected in the Q bands region, between 450 and 650 nm, and compounds were added at a final concentration of 1 mM and incubated for 2 h at 4 °C, before the acquisition of the final spectrum (Fig. S5). These Hb absorption bands are sensitive to the redox status of the protein and thus allowed to ascertain whether the tested compounds interfere with the analysis by accelerating the oxidation of oxyHb to metHb, which in turn is susceptible to heme extraction by IsdB, thus introducing an artifact in the assay. Except for C12, C17 and C28, which however had small effects, none of the compounds significantly oxidized Hb upon a 2-h incubation.

**Figure 6:**
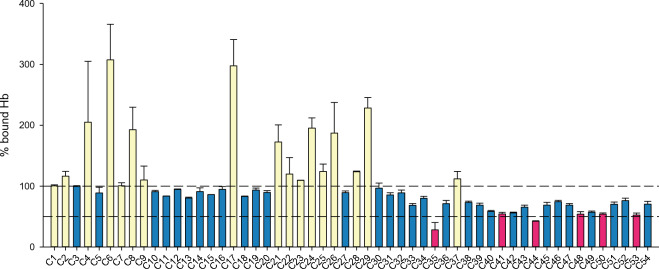
Screening of the in silico identified compounds by the IsdB-based immunoassay. Compounds, solubilized as detailed in “Materials and Methods”, were diluted to 1 mM in assay buffer and incubated for 1 h at 4 °C in the dark with 30 µM oxyHb, added to a Y165A IsdB-functionalized 96-well plate and incubated for 1 h at 4 °C in the dark. The Hb bound to IsdB was determined as detailed in “Materials and Methods”. The lines at 100% and 50%, drawn as reference, were calculated from the absorbance signal at 450 nm measured on the negative control well (i.e. oxyHb incubated without the addition of compounds). Each measurement is the average of at least 2 replicates. Yellow bars represent compounds that give a percent of bound Hb equal to or higher than 100%; blue bars represent the compounds that give a percent of bound Hb lower than 100% and higher than 55%; pink bars (percent of bound Hb lower than 55%) represents the hits selected for further characterization.

After these initial control steps, all 54 compounds were tested by the immunoassay (Fig. 6) to identify those interfering with the IsdB:Hb complex formation. Among the tested compounds, 17 were associated with an average percent of Hb bound to IsdB equal to or higher than 100% and were not further investigated (yellow bars in Fig. 6). Some of these compounds gave a signal two/threefold higher than the signal of the saturated receptor, likely as a result of interference of the compound with the HRP detection. 31 compounds showed a percentage of bound oxyHb between 55 and 100% (blue bars) and 6 compounds (C35, C41, C44, C48, C50 and C53, pink bars) gave an average percent of Hb bound to IsdB lower than 55%. C35 was peculiar because, differently from the other five compounds, gave irreproducible results in the assay. In fact, commercial C35 proved to be poorly soluble in water, and as for the other insoluble compounds the pH of the solution was adjusted with NaOH to promote solubilisation. However, this alkaline treatment led to a C35 rearrangement, which was likely the cause of the irreproducible immunoassay results.

These compounds were selected and analysed by STD-NMR to confirm their binding to Hb (Fig. S6). Hb bound to carbon monoxide (HbCO) was used in the assay in place of oxyHb as the latter is prone to spontaneously oxidize to metHb, which is paramagnetic and would cause spectral distortion. In the STD spectra of C48 and C50 no peaks were observed corresponding to the compounds (data not shown), suggesting these compounds do not interact with HbCO, at least not within the range of affinities that allow monitoring binding by this method (K_D_ in the range 10^–8^–10^–3^ M)^53^. All other compounds (C35, C41, C44 and C53) gave peaks in the STD spectra, confirming their ability to bind HbCO. However, C41, C44 and C53 had very weak peaks (i.e. a low signal-to-noise ratio), possibly indicating a low affinity, close to the upper millimolar limit of the technique. C35, instead, gave a STD spectrum with sharper peaks (Fig. S6), pointing to a higher affinity to Hb.

According to molecular docking prediction, C35, the best performing compound, when binding to Hb might interfere with the binding of the hydrophobic side chain of IsdB Phe242 and might compete with IsdB Glu247 for an electrostatic interaction with Hb Lys16. Similarly, C35 might interfere with the same interactions established by Ile447 and Glu449 in IsdH^N2^ with Hb (Fig. S7).

### Characterization of the best hits and their derivatives

**Figure 7:**
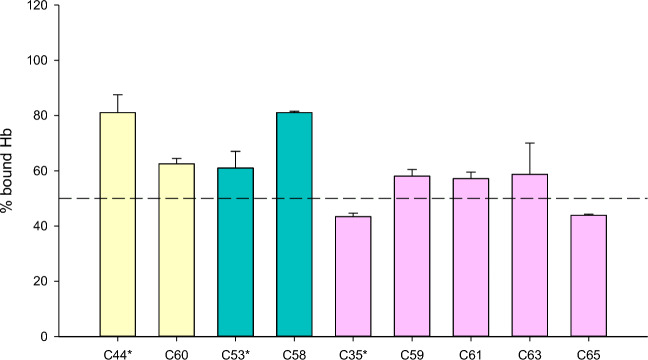
Percent of Hb bound to IsdB Y165A in the presence of 1 mM in-house synthesized compounds. Compounds, solubilized as detailed in “Materials and Methods”, were diluted to 1 mM in assay buffer and incubated for 1 h at 4 °C in the dark with 30 µM oxyHb and then added to a Y165A IsdB-functionalized 96-well plate and incubated for 1 h at 4 °C in the dark. The Hb bound to IsdB was determined as detailed in “Materials and Methods”. Each measurement is the average of at least two replicates. Compounds indicated with (*) are commercial compounds identified in the initial screening and resynthesized in-house. Color code indicates derivatives from a common parent compound indicated with the asterisk.

Among the four compounds for which the binding to Hb was confirmed by STD-NMR, three were resynthesized in-house (i.e. C35*, C44*, and C53*, the asterisk is used hereafter to indicate resynthesized compounds) and the immunoassays were repeated (Fig. 7). C41 was not further investigated because STD-NMR demonstrated that only a methyl group was binding to Hb (Fig. S6). C44* was difficult to dissolve in DMSO, probably because of stacking interactions among molecules. The synthesized molecule likely crystallizes in a different form causing a different solubility compared to the commercial compound. In the immunoassay, C44* and C53* produced a percentage of IsdB-bound Hb above 60%, while the most promising compound was C35*, giving a bound Hb percentage of ~ 40%. The preparation of concentrated stocks of C35* in DMSO solved the problem of irreproducibility observed for commercial C35, which was due to the compound undergoing a rearrangement in alkaline aqueous solution.

We then synthesized two series of C35 analogues to improve stability and/or solubility (Table 3). The first series (C59 and C61) was synthesized to increase C35 stability. C59 is the modified version of C35 in which the two protons of the α-carbon of the carbonyl group are replaced with two methyl groups. C61 is C35 with a methyl group on the indole ring. The second series (C63 and C65) was synthesized to increase C35 solubility. C63 is a modified version with an azaindole ring replacing the indole of C35. C65 has a -CH_2_COOH substituent on the indole ring.

**Table 3:** Properties of C35, C44 and C53 synthesized derivatives.

ID	Parent compound	Structure	MW
C58	C53		292.2
C59	C35		422.5
C60	C44		275.3
C61	C35		408.4
C63	C35		395.4
C65	C35		452.4

Since also C53 and C44 gave solubility issues, we prepared analogues named C58 and C60. C58 was obtained by hydrolysing the methyl ester function of C53. C60 is an analogue of C44 with a methyl substituent on the terminal -NH_2_ group of the urea substructure (Table 3). Unfortunately, the changes we made did not improve the solubility of the compounds. Thus, poorly soluble molecules bearing a carboxylic moiety (C59, C61, C63, C65) were synthesized as TRIS salts. Overall, the derivatives did not show significant improvements with respect to the parent compounds (Fig. 7).

**Figure 8:**
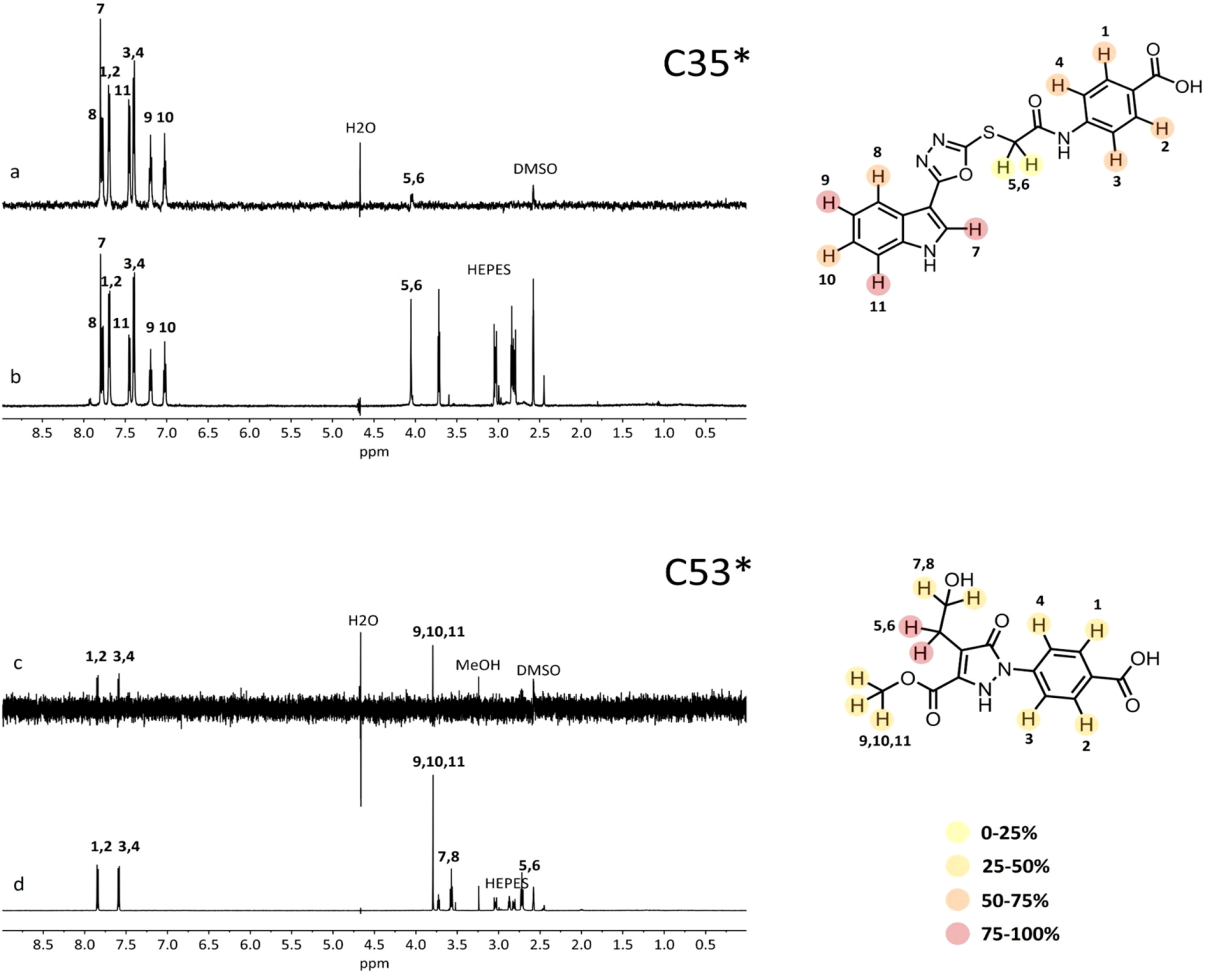
STD spectra (**a**,**c**) and off resonance spectra (**b**,**d**) of in-house synthesized compounds C35*and C53*. Resonance assignment is reported in the spectra according to the proton numbers in the structures on the right. GEM analysis results are shown with colored circles on the protons, indicating for each proton the relative % of intensity compared to the most intense signal (set as 100%). The legend indicates weak (light yellow, 0–25%), intermediate (dark yellow, 25–50%), strong (orange, 50–75%) and very strong (red, 75–100%) intensities. The contribution of HEPES buffer present in the HbCO sample could be observed around 2.5–3 ppm.

STD-NMR was repeated for C35* and C53* to confirm the data obtained for the commercial compounds. Samples were dissolved in D_6_-DMSO to prepare concentrated stocks of the compounds. First, we observed peak changes in the NMR spectra of C35 compared to C35*, confirming the rearrangement of commercial C35 in alkaline solutions. Anyway, STD-NMR spectra for C35* and C53* confirmed binding to HbCO (Fig. 8). Group epitope mapping (GEM) was performed on STD-NMR spectra of C35* and C53*. This analysis allowed to determine which are the molecule protons closer to the HbCO surface. It appeared that both the benzenic and indolic aromatic rings of C35* are involved in the interaction with HbCO. The GEM analysis for C53* indicated that the –CH_2_– protons linked to the pyrazolone-ring are those in closer contact with the protein surface, while all other not-exchangeable protons show a GEM value below 50%, pointing to a higher distance from the interaction interface.

### The best hit, C35*, has low micromolar affinity to Hb

**Figure 9:**
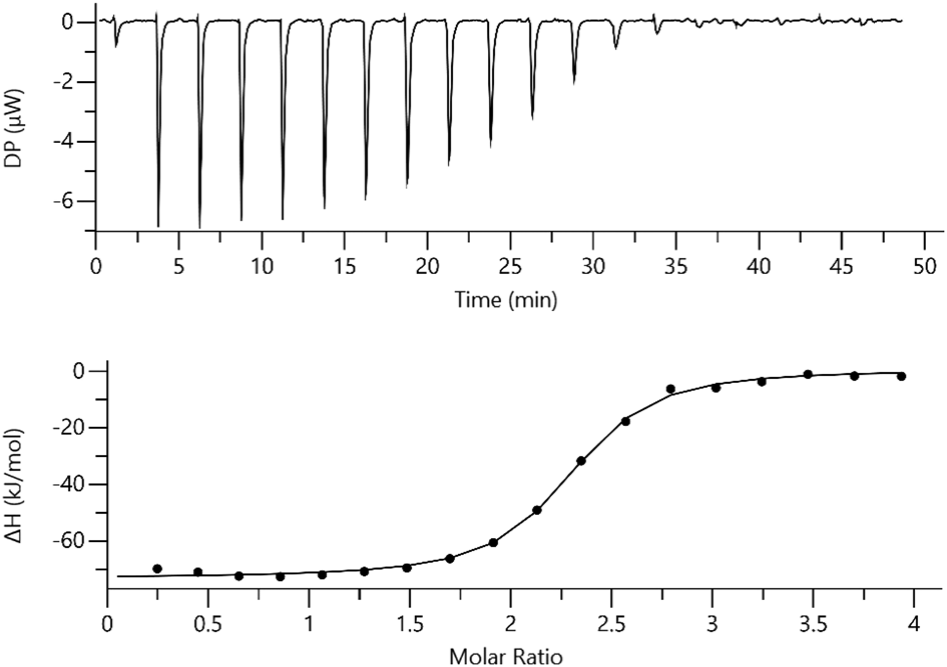
ITC experiment for HbCO (25 µM Hb tetramer) titration with C35* (500 µM). Raw data for ITC titration are shown in the upper panel, the binding isotherm of the integrated titration curve is reported in the bottom panel. The dilution heat from the control experiment was subtracted from the titration in the binding isotherm. Experiments were carried out at 25 °C in 50 mM HEPES buffer, pH 7.6. The fitting for this measurement gave the following parameters: K_D_ = 0.55 ± 0.04 µM; n = 2.21 ± 0.01; ΔH = − 73.7 ± 0.52 kJ/mol. The experiment was performed in triplicate (other two replicates in Fig. S8).

To further investigate the binding to Hb of the best hit-compound, C35*, we performed isothermal titration calorimetry to determine its binding affinity to Hb. One representative ITC titration of HbCO with C35* is reported in Fig. 9. The experiment was acquired in triplicate (other two replicates in Fig. S8A,B). The fitting of the curves with a model having one set of identical sites gave an average K_D_ of 0.57 ± 0.06 µM. The reaction stoichiometry indicates an average n value of 2.41 ± 0.16, pointing to the presence of two binding sites for C35* on each Hb tetramer. Given the symmetry of the Hb tetramer, it is plausible that C35* binds in two symmetrical sites of the protein, one on each αβ Hb dimer, in agreement with the targeted pocket shown in Fig. 1. The binding process is exothermic, with an average ΔH of -70.9 ± 2.42 kJ/mol. In conclusion, ITC confirmed that C35* binds to Hb as indicated by the immunoassay and STD-NMR.

## Conclusions

MRSA and VRSA strains represent, with other pathogens, a high-priority target, as recently stressed by the WHO, for which new antimicrobials are urgently needed. Iron restriction has been already exploited as a strategy to contrast *S. aureus* virulence but targeting PPIs to hamper the bacterial extraction of heme from Hb by hemophores has not yet been pursued.

Here we applied an integrated in silico*/*in vitro procedure for identifying disruptors of the IsdB:Hb interaction, likely able to prevent the heme transfer that follows stabilization of the aforementioned complex. By means of structure-based computations, we have screened large virtual libraries of commercial compounds and selected the most promising, based on their estimated binding mode at the Hb surface, known to be recognized by IsdB. The compounds’ capability of preventing Hb binding by IsdB was tested by a newly developed immunoassay. Most active molecules were validated by STD-NMR, resynthesized, and modified to produce derivatives. The best hit was submitted to ITC analysis to measure the binding affinity towards Hb. Following the described pipeline, we have been able to identify IsdB:Hb complex disruptors, one of which was confirmed as capable of binding Hb in the low micromolar range, and can be considered a promising lead compound for further SAR studies, if crystal structures of its complex with Hb will be obtained.

Although we are aware of the challenge of developing PPI polar inhibitors, because of the difficulty of targeting an exposed shallow surface on Hb that is mainly recognized by IsdB through hydrophobic contacts, the results demonstrate that this approach can successfully lead to identify and validate in vitro inhibitors of IsdB:Hb interaction. This could pave the way to the discovery of new antimicrobial entities exploiting an unexplored mechanism of action, and therefore likely to be less prone to induce resistance. Our future goal is to further optimize the lead compounds to increase their potency and allow molecule testing on *S. aureus* cultures.

### Supplementary Information

Supplementary Information.

## Data Availability

The datasets used and/or analysed during the current study available from the corresponding author on reasonable request.
