# Supplementary material for: Identification of small molecules affecting the interaction between human hemoglobin and Staphylococcus aureus IsdB hemophore
Source: Sci Rep. 2024 Apr 9;14:8272. doi: 10.1038/s41598-024-55931-8 (PMC11003968; doi:10.1038/s41598-024-55931-8)
Supplement: Supplementary file 1 — Supplementary Information. [file 41598_2024_55931_MOESM1_ESM.pdf]

Supplementary Information

Identification of small molecules affecting the interaction between human hemoglobin and *Staphylococcus aureus* IsdB hemophore

Monica Cozzi[1]#, Mariacristina Failla[2]#, Eleonora Gianquinto[2], Sandra Kovachka[2,3], Valeria Buoli Comani[1], Carlotta Compari[1], Omar De Bei[4], Roberta Giaccari[1], Francesco Marchesani[4], Marialaura Marchetti[4], Luca Ronda[4,5], Barbara Rolando[2], Massimo Baroni[6], Gabriele Cruciani[7], Barbara Campanini[1], Stefano Bettati[4,5], Serena Faggiano[1,5]*, Loretta Lazzarato[2], Francesca Spyrakis[2]*

[1] Department of Food and Drug, University of Parma, Parma, Italy
[2] Department of Drug Science and Technology, University of Turin, Turin, Italy
[3] The Herbert Wertheim UF Scripps Institute for Biomedical Innovation & Technology, Jupiter, Florida, USA
[4] Department of Medicine and Surgery, University of Parma, Parma, Italy
[5] Institute of Biophysics, National Research Council, Pisa, Italy
[6] Molecular Discovery Ltd, Kinetic Business Centre, Elstree, Borehamwood, Hertfordshire, UK
[7] Department of Chemistry, Biology and Biotechnology, University of Perugia, Perugia, Italy

# these authors contributed equally to this work

* corresponding authors

Table of Contents

Figure S1. development of an immunoassay for IsdB:Hb complex formation S3

Chemical Synthesis details S4

Figure S2. IsdB^{N1} and IsdH^{N2} binding to Hb alpha S9

Figure S3. Superposition of Hp:Hb to IsdB:Hb complex S10

Figure S4. Visible spectra of commercial compounds at 1 mM concentration S11

Figure S5. Spectral comparison of Hb Q bands before (grey lines) and after (red lines) incubation with each commercial compound S12

Figure S6. STD-NMR spectra S13

Figure S7. Potential of compound 35 to inhibit the Isd-Hb interaction S14

Figure S8. ITC experiments S15

References S16

Figure S1. Development of an immunoassay for IsdB:Hb complex formation. **Panel A**. The optimal antibody concentration was evaluated using 2 pmol StrepTag®II-Y165A IsdB attached on the bottom of the microplate and 30 μM oxyHb solution to sub-saturate IsdB. Four HRP-conjugated anti-Hb polyclonal antibody dilutions were tested. **Panel B.** Effect of different Ab dilutions on the Hb binding to Y165A IsdB. 2 pmol StrepTag®II-Y165A IsdB was attached on the bottom of the microplate and different concentrations of oxyHb solutions were added. Three different Ab dilutions were used for detection. Lines through data points are the fitting to equation 1. **Panel C.** The effect of incubation time of oxyHb with IsdB-functionalized plate was assessed between 1 and 3 hours using 2 pmol StrepTag®II-Y165A IsdB attached on the bottom of the microplate, 30 μM oxyHb solution and 1:1,000 Ab dilution. **Panel D.** The stability of the IsdB:Hb complex upon plate washing was assessed using 2 pmol StrepTag®II-Y165A IsdB attached on the bottom of the microplate, 30 μM oxyHb solution and 1:1,000 diluted Ab. The number of washings is reported for each step of the procedure: after IsdB functionalization, after Hb incubation for 1 h and after Ab incubation. The reference is the procedure with three washings for each step.

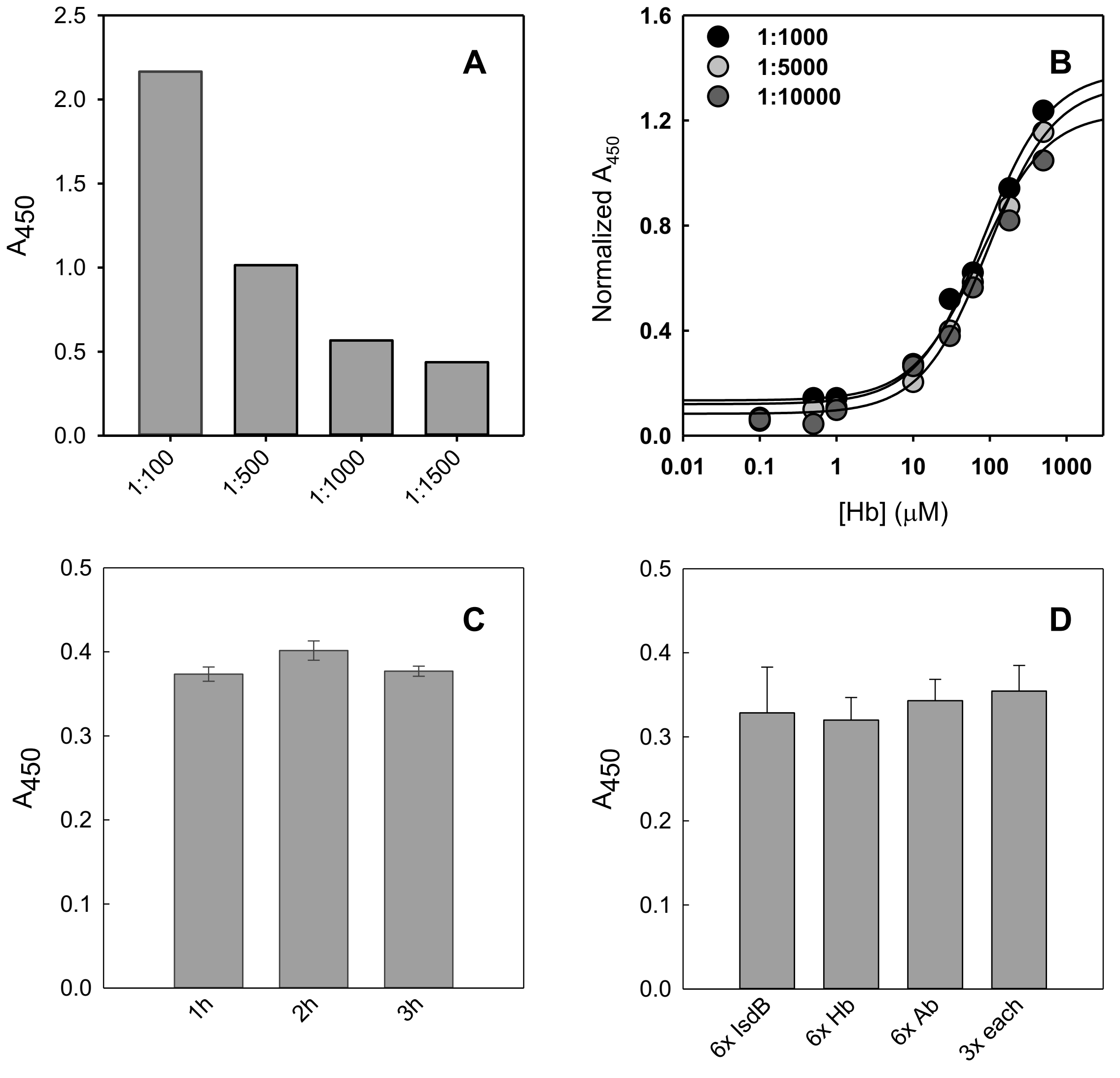

Chemical synthesis details.

1H-Indole-3-carbohydrazide (1) was synthesized according to literature [1].

1-Methyl-1H-indole-3-carbohydrazide (2) was synthesized according to literature [2].

Potassium 2-(3-(methoxycarbonyl)-1H-indol-1-yl)acetate (11) and 2-(3-(hydrazinecarbonyl)-1H-indol-1-yl)acetic acid (3). was synthesized according to literature [3].

1*H*-Pyrrolo[2,3-b]pyridine-3-carbohydrazide (4) and **5-(1*H*-pyrrolo[2,3-b]pyridin-3-yl)-1,3,4-oxadiazole-2-thiol** (**8**) were synthesised according to literature [4].

5-(1H-Indol-3-yl)-1,3,4-oxadiazole-2-thiol (5) was synthesized according to literature [5].

5-(1-Methyl-1H-indol-3-yl)-1,3,4-oxadiazole-2-thiol (6). To a solution of **2** (1.1 g, 5.82 mmol) in EtOH (20 mL) 85% KOH (0.614 g, 9.31 mmol) was added and the mixture was stirred for 30 min at room temperature, after that, CS_2 (0.563 mL, 9.31 mmol) was added and the reaction mixture was refluxed for 4 h. Upon completion, the solvent was removed in vacuum and the yellow residue was dissolved in water (3 mL) and the solution acidified to pH 2 adding with 1 M HCl. The precipitate was filtered, collected and purified by silica gel column chromatography with PE/EtOAc (90/10 v/v) as the eluent. The desired compound was obtained as a yellow solid (54 % yield). ^{1}H NMR (600 MHz, DMSO-d_6) δ 8.22 (s, 1H), 7.91 (d, J = 7.9 Hz, 1H), 7.61 (d, J = 7.9 Hz, 1H), 7.42 – 7.15 (m, 2H), 3.90 (s, 3H). ^{13}C NMR (150 MHz, DMSO-d_6) δ 175.9, 158.7, 137.1, 132.5, 123.8, 123.1, 121.7, 120.0, 111.1, 97.3, 33.2. ESI-MS $[M-H]^-$: *m/z* 230.3.

2-(3-(5-Mercapto-1,3,4-oxadiazol-2-yl)-1*H*-indol-1-yl)acetic acid (7). To a solution of **3** (0.336 g, 1.44 mmol) in EtOH (25 mL) 85% KOH (0.247 g, 3.744 mmol) was added and the mixture was stirred for 30 min at r.t., after that, CS_2 (0.14 mL, 2.304 mmol) was added, and the reaction mixture was refluxed for 4 h. Upon completion, the solvent was removed in vacuum and the yellow residue was dissolved in water (3 mL) and the solution acidified to pH 1 with 1 M HCl. The precipitate was filtered, collected and purified by silica gel chromatography with CH_2Cl_2/acetone/HCOOH (95/5/0.1 v/v/v) as the eluent. **13** was obtained as a white solid in 36% yield. ^{1}H NMR (600 MHz, CD_3OD) δ 8.02 (d, J = 8.0 Hz, 1H), 7.96 (s, 1H), 7.44 (d, J = 8.0 Hz, 1H), 7.34 – 7.32 (m, 1H), 7.27 – 7.25 (m, 1H), 5.10 (s, 2H). ^{13}C NMR (150 MHz, CD_3OD) δ 171.4, 160.6, 138.7, 132.8, 125.7, 124.7, 122.9, 121.8, 111.4, 100.6, 48.4. ESI-MS $[M-H]^-$: *m/z* 274.3.

4-(2-Bromoacetamido)benzoic acid (9) was synthesized according to literature [6].

4-(2-Bromo-2-methylpropanamido)benzoic acid (10). was synthesized according to literature [7].

4-(2-((5-(1H-indol-3-yl)-1,3,4-oxadiazol-2-yl)thio)acetamido)benzoic acid (C35). To a solution of **5** (0.623 g, 2.9 mmol) in acetone (85 mL) K_2CO_3 (0.793 g, 5.8 mmol) and **9** (0.598 g, 2.32 mmol) were added and the reaction was stirred at room temperature for 30 min and then refluxed for 3 h. Upon completion, the mixture was diluted with H_2O (50 mL) and washed with CH_2Cl_2 (3

× 20 mL); then the aqueous phase was acidified with 1 M HCl, and the formed precipitate was filtered and crystallized from a 1/1 EtOH/H_2O mixture to obtain **C35** as a white solid in 29% yield. ^{1}H NMR (600 MHz, DMSO-d_6) δ 12.76 (s, 1H), 12.03 (bs, 1H), 10.76 (s, 1H), 8.13 (d, J = 2.9 Hz, 1H), 8.02 (d, J = 8.0 Hz, 1H), 7.92 (d, J = 8.8 Hz, 2H), 7.72 (d, J = 8.8 Hz, 2H), 7.52 (d, J = 8.2, 1H), 7.29 – 7.21 (m, 1H), 7.22 – 7.12 (m, 1H), 4.36 (s, 2H). ^{13}C NMR (150 MHz, DMSO-d_6) δ 166.4, 165.2, 162.7, 159.8, 142.3, 136.0, 130.1, 127.8, 125.2, 123.4, 122.5, 120.8, 119.7, 118.1, 112.0, 98.6, 36.5. ESI-MS [M-H]$^-$: *m/z* 393.5.

4-(2-((5-(1H-Indol-3-yl)-1,3,4-oxadiazol-2-yl)thio)-2-methylpropanamido)benzoic acid (C59). To a solution of **5** (0.217 g, 1 mmol) in acetone (30 mL) K_2CO_3 (0.276 g, 2 mmol) and **10** (0.286 g, 1 mmol) were added and the reaction was stirred at room temperature for 30 min and then refluxed for 18 h. Upon completion, the mixture was diluted with H_2O (50 mL) and washed with CH_2Cl_2 (3 × 20 mL); then the aqueous phase was acidified with 1 M HCl and extracted with EtOAc (2 × 20 mL). The combined organic phases were dried over Na_2SO_4 and concentrated to dryness. The crude product was washed with acetone, filtered, and dried under vacuum to yield **C59** (75 %). ^{1}H NMR (600 MHz, DMSO-d_6) δ 11.62 (s, 1H), 10.19 (s, 1H), 8.11 (d, J = 8.4 Hz, 2H), 8.07 – 8.06 (m, 1H), 7.60 (d, J = 8.4 Hz, 2H), 7.42 (m, 1H), 7.16 – 7.09 (m, 2H), 2.08 (s, 1H), 1.73 (s, 6H). ^{13}C NMR (150 MHz, DMSO-d_6) δ 176.6, 168.6, 166.7, 162.3, 138.9, 135.9, 131.0, 130.1, 128.7, 126.2, 125.0, 122.1, 120.9, 120.6, 111.9, 89.6, 30.7, 27.9. ESI-MS [M-H]$^-$: *m/z* 421.5.

4-(2-((5-(1-Methyl-1H-indol-3-yl)-1,3,4-oxadiazol-2-yl)thio)acetamido)benzoic acid (C61). To a solution of **6** (0.500 g, 2.16 mmol) in acetone (70 mL) K_2CO_3 (0.598 g, 4.32 mmol) and **9** (0.558 g, 2.16 mmol) were added and the reaction was stirred at room temperature for 30 min and then refluxed for 3 h. Upon completion, the mixture was diluted with H_2O (50 mL) and washed with CH_2Cl_2 (3 × 20 mL); then the aqueous phase was acidified with HCl 1M, and extracted with EtOAc (2 × 20 mL). The combined organic phases were dried over Na_2SO_4 and concentrated to dryness. The crude product was crystallized from MeOH to give the final compound as a white solid (33% yield). ^{1}H NMR (600 MHz, DMSO-d_6) δ 10.75 (s, 1H); 8.14 (s, 1H); 8.01 (d, J = 7.8 Hz, 1H); 7.92 (d, J = 9 Hz, 2H); 7.72 (d, J = 9 Hz, 2H), 7.59 (d, J = 8.4 Hz, 1H), 7.32 – 7.21 (m, 2H), 4.35 (s, 2H), 3.88 (s, 3H). ^{13}C NMR (150 MHz, DMSO-d_6) δ 166.9, 165.7, 162.9, 160.3, 142.7, 137.0, 131.9, 130.6, 125.6, 124.2, 123.0, 121.6, 120.3, 118.5, 111.0, 98.0, 37.0, 33.1. ESI-MS [M-H]$^-$: *m/z* 407.3.

4-(2-((5-(1H-Pyrrolo[2,3-b]pyridin-3-yl)-1,3,4-oxadiazol-2-yl)thio)acetamido)benzoic acid (C63). To a solution of **8** (0.430 g, 1.97 mmol) in CH_3CN (30 mL) K_2CO_3 (0.550 g, 3.94 mmol) and **9** (0.510 g, 1.97 mmol) were added and the reaction was refluxed for 18 h. Upon completion, the solvent was partially removed in vacuum and the mixture was diluted with H_2O (50 mL) and washed with CH_2Cl_2 (3 × 20 mL); then the aqueous phase was acidified with HCl 1M. The formed precipitate was filtered and crystallization from *i*PrOH yielded the desired compound as a white solid (17%). ^{1}H NMR (600 MHz, DMSO-d_6) δ 12.62 (bs, 1H), 10.94 (s, 1H), 8.36 (m, 2H), 8.30 (s, 1H), 7.93 – 7.88 (m, 2H), 7.76 – 7.71 (m, 2H), 7.24 (dd, J = 7.9, 4.7 Hz, 1H), 4.39 (s, 2H). ^{13}C

NMR (150 MHz, DMSO-d_6) δ 166.8, 165.7, 162.4, 160.7, 148.6, 144.5, 142.7, 130.5, 128.7, 128.5, 125.6, 118.5, 117.5, 116.3, 98.0, 36.8. $[M+H]^+$: *m/z* 396.4.

4-(2-((5-(1-(Carboxymethyl)-1H-indol-3-yl)-1,3,4-oxadiazol-2-yl)thio)acetamido)benzoic acid (C65). To a solution of **7** (0.050 g, 0.178 mmol) in CH_3CN (5 mL) K_2CO_3 (0.098 g, 0.712 mmol) and **9** (0.046 g, 0.178 mmol) were added and the reaction was refluxed for 18 h. Upon completion, the solvent was removed in vacuum and the mixture was diluted with H_2O (10 mL) and acidified with 1M HCl. The formed precipitate was filtered and washed with MeOH to give **C65** as a white solid with 80% yield. ^1H NMR (600 MHz, DMSO-d_6) δ 10.76 (s, 1H), 8.18 (s, 1H), 8.02 (d, J = 7.9 Hz, 1H), 7.91 (d, J = 8.7 Hz, 2H), 7.71 (d, J = 8.7 Hz, 2H), 7.57 (d, J = 8.3 Hz, 1H), 7.29 (t, J = 7.5 Hz, 1H), 7.22 (t, J = 7.5 Hz, 1H), 5.16 (s, 2H), 4.36 (s, 2H). ^{13}C NMR (150 MHz, DMSO-d_6) δ 169.8, 166.9, 165.7, 162.7, 160.5, 142.7, 137.0, 132.1, 130.6, 125.6, 124.1, 123.2, 121.7, 120.3, 118.5, 111.1, 98.8, 47.5, 37.0. ESI-MS $[M-H]^-$: *m/z* 451.4.

General procedure for the synthesis of C35, **C59**, **C61**, **C63** and **C65 TRIS salts.** Equimolar amounts of TRIS (tris(hydroxymethyl)aminomethane) and C-compounds were mixed in H_2O/EtOH (1/1 v/v) and maintained under vigorous stirring for 30 min. The suspended particles were filtered out and the solutions were lyophilized to obtain the corresponding TRIS salts.

***N*-Carbamoyl-4-chlorobutanamide (11) and 4-chloro-*N*-(methylcarbamoyl)butanamide (11a).** To a suspension of urea or 1-methylurea (33.33 mmol) in benzene (2.5 mL) and 2 drops of concentrated H_2SO_4, 4-chlorobutanoyl chloride (16.67 mmol) was added and the mixture was refluxed for 3 h, after which the reaction mixture was allowed to reach room temperature and was stirred for 12 h. Upon completion, the formation of a precipitate was observed. The solid was filtered and dried in vacuum to obtain the desired compounds **11** (80% yield) or **11a** (84 %).

N-Carbamoyl-4-chlorobutanamide (11). ^1H NMR (600 MHz, DMSO-d_6) δ 10.19 (s, 1H), 7.70 (bs, 1H), 7.21 (bs, 1H), 3.64 (t, J = 7.2 Hz, 2H), 2.44 (t, J = 7.2 Hz, 2H), 1.95 (quint, J = 7.2 Hz, 2H). ^{13}C NMR (150 MHz, DMSO-d_6) δ 173.9, 153.8, 44.7, 32.8, 27.1. ESI-MS $[M+Na]^+$: *m/z* 187.2/189.2.

4-Chloro-N-(methylcarbamoyl)butanamide (11a). ^1H NMR (600 MHz, DMSO-d_6) δ 10.33 (bs, 1H), 8.16 (bs, 1H), 3.63 (t, J = 7.2Hz, 2H), 2.69 (d, J = 4.7Hz, 3H), 2.43 (t, J = 7.2Hz, 2H), 1.95 (quint, J = 7.2 Hz, 2H). ^{13}C NMR (150 MHz, DMSO-d_6) δ 173.8, 153.8, 44.7, 32.8, 27.2, 25.9. ESI-MS $[M+Na]^+$: *m/z* 201.3/203.3

***N*-Carbamoyl-4-iodobutanamide (12) and 4-iodo-*N*-(methylcarbamoyl)butanamide (12a).** To a solution of **11** or **11a** (12.15 mmol) in acetone (150 mL) sodium iodide (36.45 mmol) was added and the mixture was refluxed for 8 h. Upon completion, the formed precipitate was filtered and dried in vacuum to obtain the desired compounds **12** and **12a** as white solids (62% and 79% yields).

N-Carbamoyl-4-iodobutanamide (12). ^{1}H NMR (600 MHz, DMSO-d$_6$) δ 10.20 (s, 1H), 7.70 (bs, 1H), 7.22 (bs, 1H), 3.63 (t, J = 7.2 Hz, 2H), 2.44 (t, J = 7.2 Hz, 2H), 1.95 (quint, J = 7.2 Hz, 2H). ^{13}C NMR (150 MHz, DMSO-d$_6$) δ 173.9, 153.8, 44.7, 32.8, 27.1. ESI-MS [M+Na]$^+$: *m/z* 279.4.

4-Iodo-N-(methylcarbamoyl)butanamide (12a). ^{1}H NMR (600 MHz, DMSO-d$_6$) δ 10.33 (s, 1H), 8.15 (s, 1H), 3.25 (t, J = 7.2 Hz, 2H), 2.69 (d, J = 4.7 Hz, 3H), 2.38 (t, J = 7.2 Hz, 2H), 2,00 (q, J = 7.2 Hz, 2H). ^{13}C NMR (150 MHz, DMSO-d$_6$) δ 173.4, 153.7, 36.2, 28.2, 25.9, 7.32. ESI-MS [M+H]$^+$: *m/z* 271.1.

***N*-Carbamoyl-4-thiocyanatobutanamide (13) and *N*-(methylcarbamoyl)-4-thiocyanatobutanamide (13a).** To a solution of **12** or **12a** (5.86 mmol) in acetone (50 mL) potassium thiocyanate (5.86 mmol) was added and the reaction was refluxed for 4 hours. Upon completion, the formed precipitate was isolated by filtration and dried in vacuum to obtain the desired compounds **13** and **13a** in 65% or 90% yield.

N-Carbamoyl-4-thiocyanatobutanamide (13). ^{1}H NMR (600 MHz, DMSO-d$_6$) δ 10.26 (s, 1H), 7.68 (bs, 1H), 7.24 (bs, 1H), 3.09 (t, J = 7.2 Hz, 2H), 2.46 (t, J = 7.2 Hz, 2H), 1.95 (quint, J = 7.2 Hz, 2H). ^{13}C NMR (150 MHz, DMSO-d$_6$) δ 174.2, 154.3, 114.0, 34.1, 33.2, 25.3. ESI-MS [M+Na]$^+$: *m/z* 210.4.

N-(Methylcarbamoyl)-4-thiocyanatobutanamide (13a). ^{1}H NMR (600 MHz, DMSO-d$_6$) δ 10.35 (bs, 1H), 8.16 (bs, 1H), 3.08 (t, J = 7.2 Hz, 2H), 2.69 (d, J = 4.7 Hz, 3H), 2.45 (t, J = 7.3 Hz, 2H), 1.95 (quint, J = 7.3 Hz, 2H). ^{13}C NMR (150 MHz, DMSO-d$_6$) δ 173.5, 153.7, 113.1, 33.8, 32.7, 25.9, 24.8.

4-((5-Amino-1,3,4-thiadiazol-2-yl)thio)-*N*-carbamoylbutanamide (C44) and 4-((5-amino-1,3,4-thiadiazol-2-yl)thio)-N-(methylcarbamoyl)butanamide (C60). To a solution of **13** or **13a** (3.74 mmol) in TFA (8 mL) thiosemicarbazide (11.22 mmol) was added and the reaction was refluxed for 4 h. Upon completion, the mixture was cooled at 0 °C with an ice bath and neutralized with a solution of 30% NH_3 until the formation of a precipitate was observed. The solid was isolated by filtration, washed with H_2O and EtOH and dried in vacuum to give the desired compounds **C44** and **C60** as white solids. (Yield 29% and 30%).

4-((5-Amino-1,3,4-thiadiazol-2-yl)thio)-N-carbamoylbutanamide (C44). ^{1}H NMR (600 MHz, DMSO-d$_6$) δ 10.18 (s, 1H), 7.70 (bs, 1H), 7.32 (s, 2H), 7.21 (bs, 1H), 3.03 (t, J = 7.2Hz, 2H), 2.42 (t, J = 7.2Hz, 2H), 1.86 (quint, J = 7.2Hz, 2H). ^{13}C NMR (150 MHz, DMSO-d$_6$) δ 174.0, 169.7, 153.8, 149.8, 34.2, 33.7, 24.3. ESI-MS [M+H]$^+$: *m/z* 262.4.

4-((5-Amino-1,3,4-thiadiazol-2-yl)thio)-N-(methylcarbamoyl)butanamide (C60). ^{1}H NMR (600 MHz, DMSO-d$_6$) δ 10.30 (bs, 1H), 8.16 (bs, 1H), 7.30 (s, 2H), 3.03 (t, J = 7.2 Hz, 2H), 2.69 (d, J = 4.7 Hz, 3H), 2.41 (t, J = 7.3 Hz, 2H), 1.86 (quint, J = 7.3 Hz, 2H). ^{13}C NMR (150 MHz, DMSO-d$_6$) δ 173.9, 169.6, 153.8, 149.7, 34.1, 33.7, 25.9, 24.4. ESI-MS [M+H]$^+$: *m/z* 276.4.

4-Hydrazineylbenzoic acid and methyl 2-oxo-2-(2-oxotetrahydrofuran-3-yl)acetate were synthesized according to literature [8,9].

4-(4-(2-Hydroxyethyl)-3-(methoxycarbonyl)-5-oxo-2,5-dihydro-1H-pyrazol-1-yl)benzoic acid (C53). To a solution of methyl 2-oxo-2-(2-oxotetrahydrofuran-3-yl)acetate (0.800 mg, 4.65 mmol) in MeOH (160 mL) was added 4-hydrazinobenzoic acid (4.3 g, 23.25 mmol), triethylamine (3.23 mL, 23.25 mmol), and a catalytic amount of acetic acid. The solution was stirred at room temperature for 24 h. Upon completion, the solvent was removed in vacuum and the residue was purified by silica gel column chromatography with CH_2Cl_2/MeOH (90/10 v/v) as the eluent and gets the desired compound as a white solid (0.515 g, 37% yield). ^{1}H NMR (600 MHz, DMSO-d_6) δ 8.23 – 7.99 (m, 2H), 7.92 (d, J = 8.7 Hz, 2H), 3.80 (s, 3H), 3.55 (t, J = 7.0 Hz, 2H), 2.82 (t, J = 7.0 Hz, 2H). ^{13}C NMR (150 MHz, DMSOd$_6$) δ 166.7, 163.0, 154.0, 141.7, 140.6, 140.4, 130.4, 128.7, 121.3, 61.3, 51.6, 25.5. ESI-MS [M-H]$^-$: *m/z* 305.3.

1-(4-Carboxyphenyl)-4-(2-hydroxyethyl)-5-oxo-2,5-dihydro-1H-pyrazole-3-carboxylic acid (C58). To a solution of **C53** (0.100 g, 0.299 mmol) in MeOH (8 mL) 1 M NaOH (2 mL) was added and the resultant mixture was stirred at room temperature for 12 h. Then the MeOH was removed under reduced pressure. The residue was diluted with H_2O (15 mL) and acidified to pH 1 with 1 M HCl. The light orange precipitate was filtered, washed with H_2O and CH_2Cl_2, collected, and dried under vacuum (0.077 g, 88%). ^{1}H NMR (600 MHz, DMSO-d_6) δ 12.96 (bs, 1H), 8.23 – 7.99 (m, 2H), 7.92 (d, J = 8.7 Hz, 2H), 3.55 (t, J = 7.0 Hz, 2H), 2.82 (t, J = 7.0 Hz, 2H). ^{13}C NMR (150 MHz, DMSO-d_6) δ 166.7, 163.9, 151.5, 1482, 141.8, 141.6, 130.4, 128.5, 121.3, 61.2, 25.7. ESI-MS [M-H]$^-$: *m/z* 291.3

Figure S2. IsdB^{N1} (A) and IsdH^{N2} (B) binding to Hbα (PDB ID 5VMM and 4XS0, respectively). Proteins are shown as cartoon, the groove used for virtual screening as green contour. Residues of IsdB^{N1} or IsdH^{N2} at the interface are shown as sticks and labelled according to their PDB numbering. IsdB residues are labelled in blue, IsdH residues are labelled in yellow, Hbα residues are labelled in magenta, and Hbβ residues are labelled in teal. Y165 in IsdB is highlighted in lilac, while Y366 in IsdH is highlighted in orange.

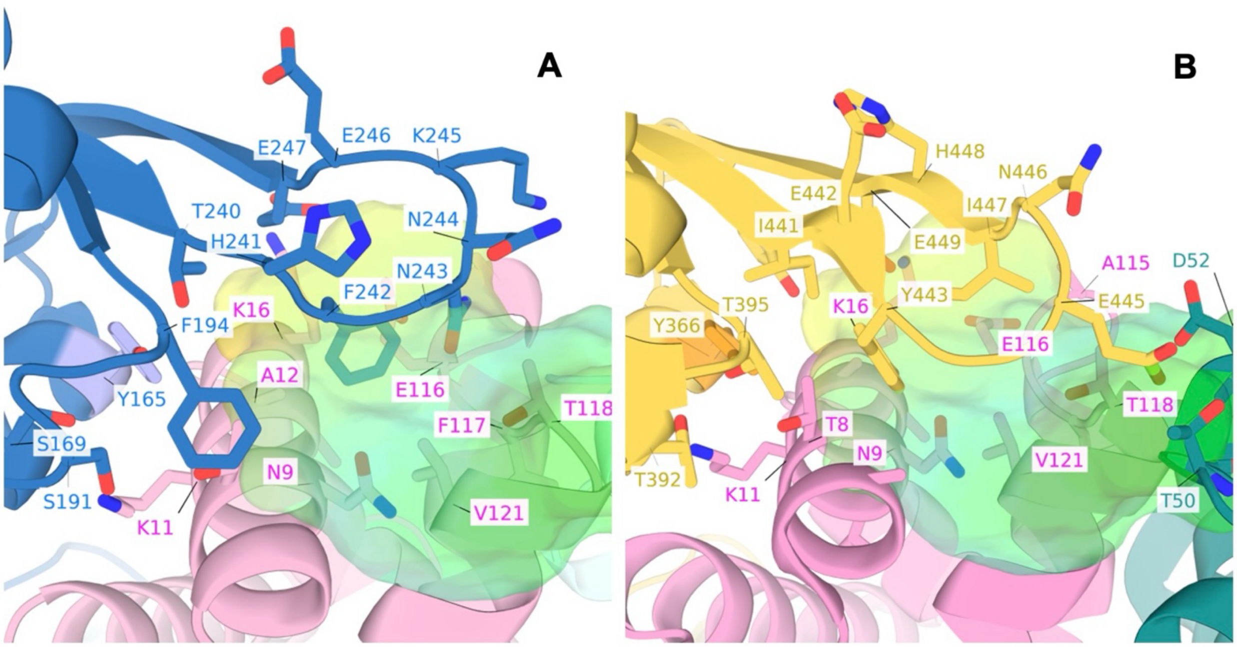

Figure S3. Superposition of Hp:Hb complex (PDB ID 4X0L) to IsdB:Hb complex (PDB ID 5VMM). The proteins are shown in cartoons (IsdB: blue; Hbα: pink; Hbβ: light blue, Hp: orange), the targeted pocket as light green-yellow contour and the heme groups in yellow capped sticks.

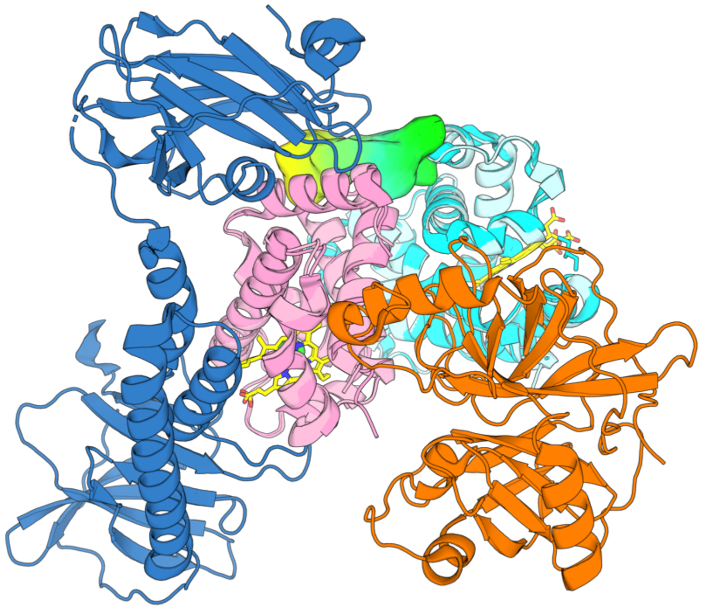

Figure S4. Visible spectra of commercial compounds at 1 mM concentration. The yellow line is a reference at 450 nm.

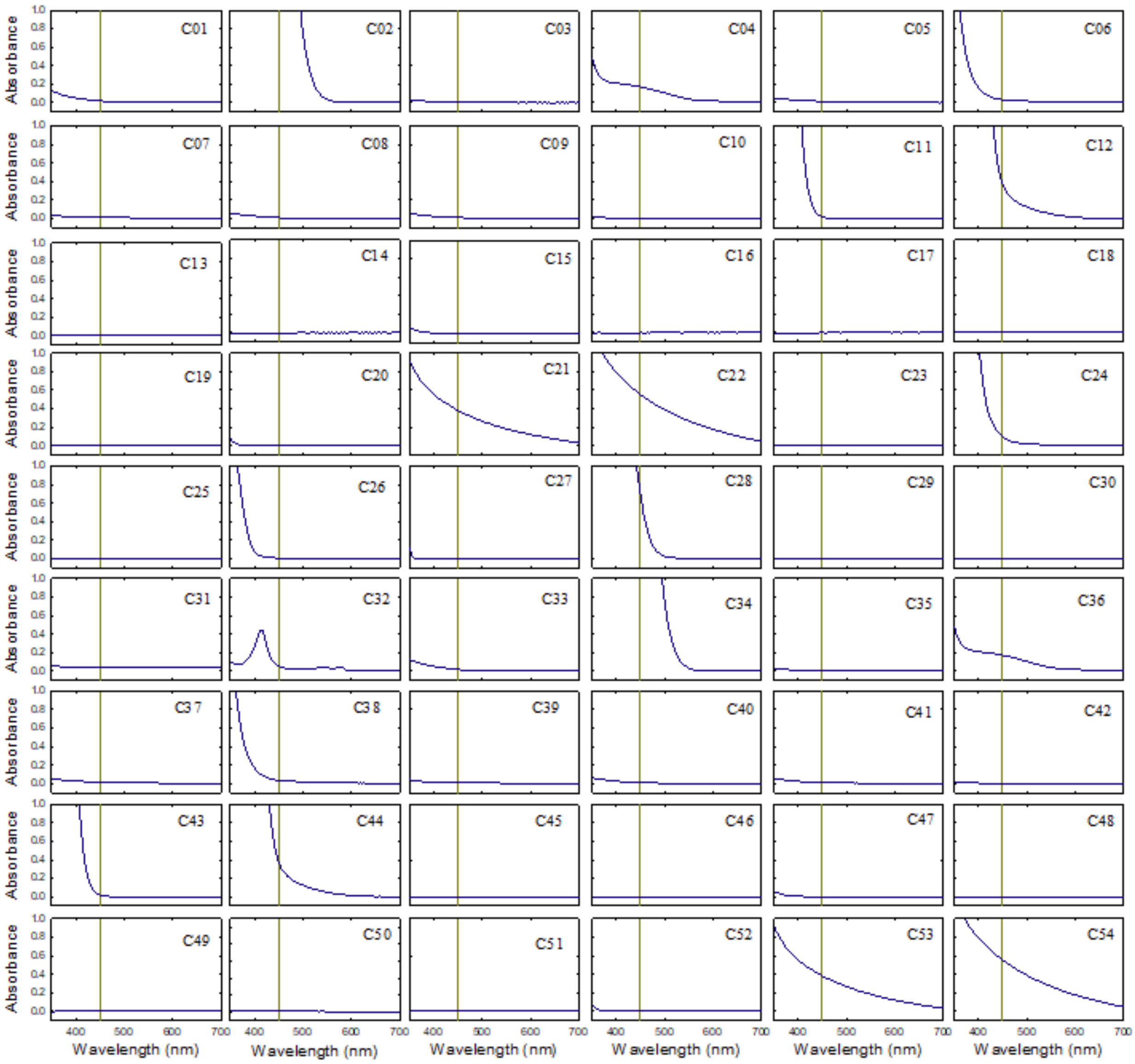

Figure S5. Spectral comparison of Hb Q bands before (grey lines) and after (red lines) incubation with each commercial compound. The spectra of Hb after incubation were subtracted by the signal of the compound alone in solution.

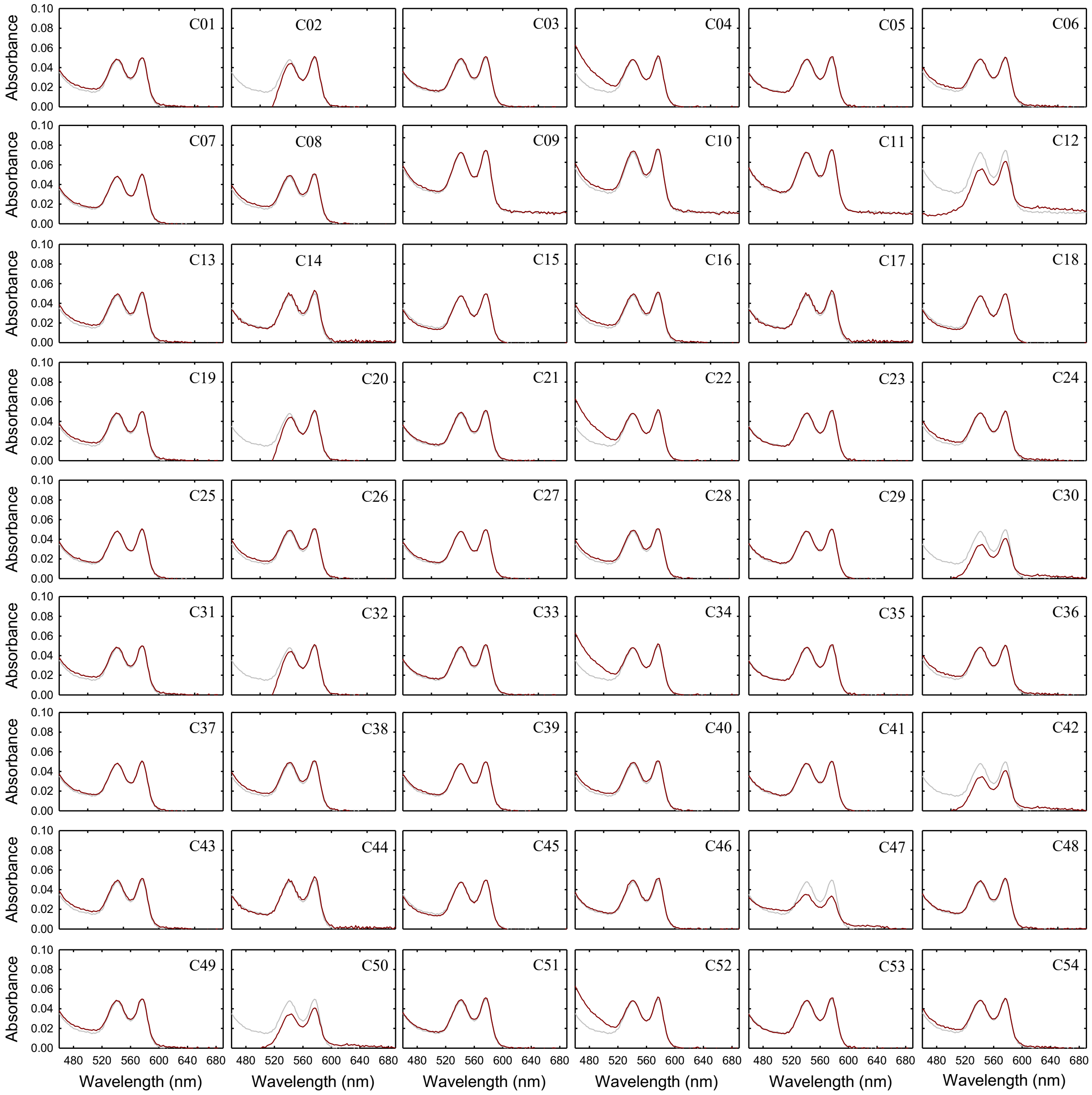

Figure S6. STD-NMR spectra (top, for each compound) and off-resonance spectra (bottom, for each compound) for the commercial compounds C35, C41, C44, and C53. Blue arrows indicate the positions of the peaks in the STD spectra; blue circles show the corresponding protons in the structures.

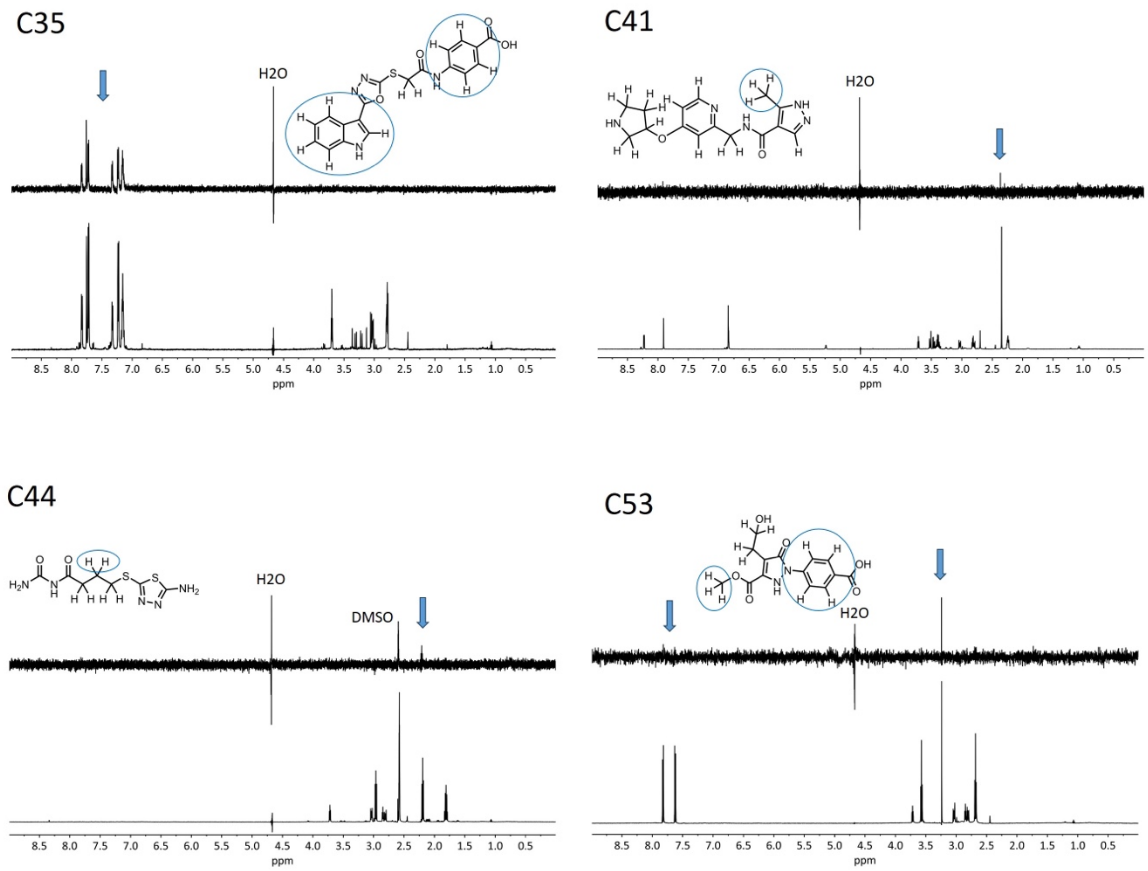

Figure S7. Potential of compound C35 to inhibit the Isd:Hb interaction. Docking pose of C35 aligned to (**A**) IsdB^{N1} in PDB 5VMM (blue) and (**B**) IsdH^{N2} in 4XS0.

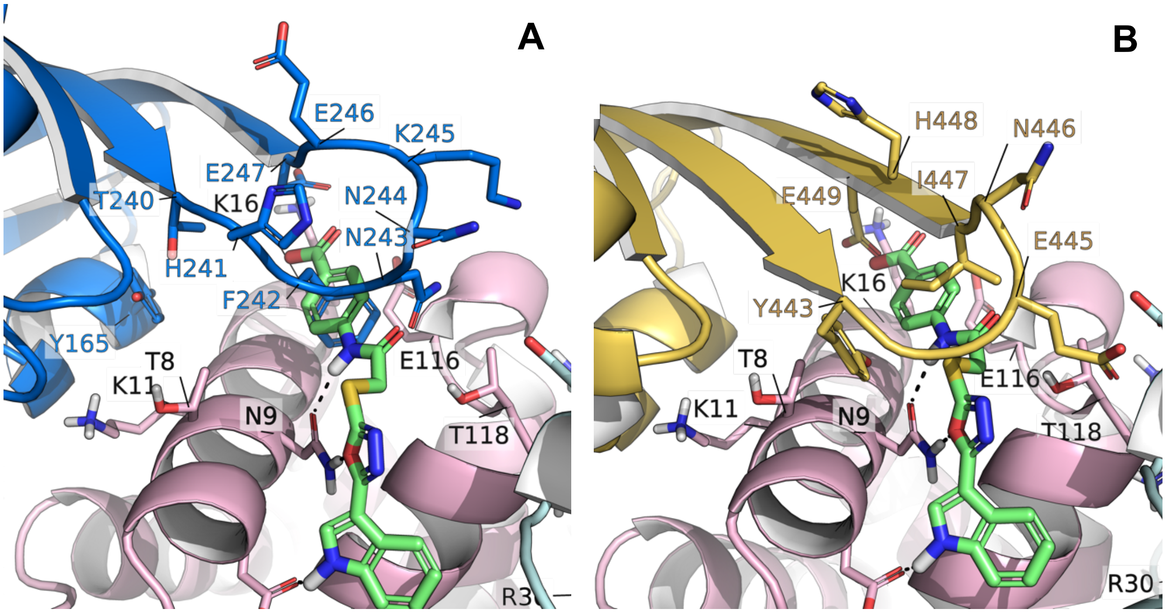

Figure S8: **A, B: ITC experiments for HbCO (25 µM Hb tetramer) titration with C35* (500 µM)** (here are reported the other two measurements in the triplicate, first measurement reported in Figure 9, main text). Raw data for ITC titration are shown in the upper panels, the binding isotherm of the integrated titration curve are reported in the bottom panels. The dilution heat from the control experiment was subtracted from the titration in the binding isotherm. Experiments were carried out at 25 °C in 50 mM HEPES buffer, pH 7.6. The fitting for experiment in panel A gave the following parameters: $K_D = 0.48 \pm 0.07$ µM; $n = 2.31 \pm 0.02$; $\Delta H = -73.0 \pm 0.78$ kJ/mol. The fitting for experiment in panel B gave the following parameters: $K_D = 0.68 \pm 0.05$ µM; $n = 2.72 \pm 0.01$; $\Delta H = -66.1 \pm 0.39$ kJ/mol. **C. control experiment** in which the reaction cell was filled with the buffer solution with 0.5% DMSO only, while the syringe was filled with 500 µM C35*.

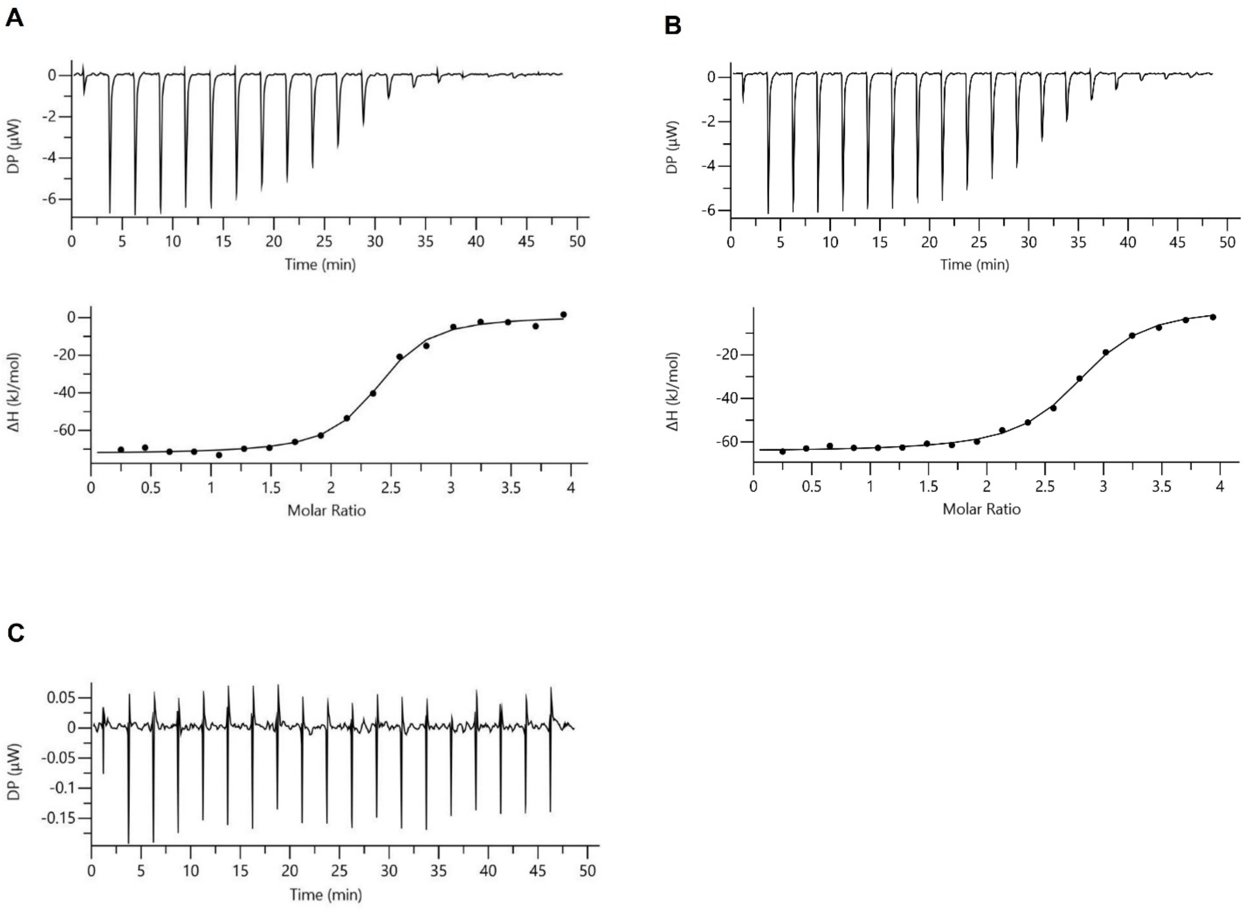

References

1. Zhang, M.-Z. *et al.* Synthesis and antifungal activity of 3-(1,3,4-oxadiazol-5-yl)-indoles and 3-(1,3,4-oxadiazol-5-yl)methyl-indoles. *Eur. J. Med. Chem.* **63**, 22–32 (2013).
2. Yang, F. *et al.* Discovery of new indole-based 1,2,4-triazole derivatives as potent tubulin polymerization inhibitors with anticancer activity. *New J. Chem.* **45**, 21869–21880 (2021).
3. Dak, M. *et al.* Novel heterocyclic hydroxamates as inhibitors of the mycobacterial zinc metalloprotease Zmp1 to probe its mechanism of function. *Eur. J. Med. Chem.* **244**, 114831 (2022).
4. Allegretti, M. *et al.* A Practical Synthesis of 7-Azaindolylcarboxy-endo-tropanamide (DF 1012). *Org. Process Res. Dev.* **7**, 209–213 (2003).
5. Song, Z.-L. *et al.* Diversity-oriented synthesis and antifungal activities of novel pimprinine derivative bearing a 1,3,4-oxadiazole-5-thioether moiety. *Mol. Diver.s* **25**, 205–221 (2021).
6. Park, D. *et al.* Noninvasive Imaging of Cell Death Using an Hsp90 Ligand. *J. Am. Chem. Soc.* **133**, 2832–2835 (2011).
7. Sugnaux, C., Lavanant, L. & Klok, H.-A. Aqueous Fabrication of pH-Gated, Polymer-Brush-Modified Alumina Hybrid Membranes. *Langmuir* **29**, 7325–7333 (2013).
8. Park, J. H., Manivannan, R., Jayasudha, P. & Son, Y.-A. Spontaneous optical response towards cyanide ion in water by a reactive binding site probe. *Spectrochimica Acta Part A: Molecular and Biomolecular Spectroscopy* **233**, 118190 (2020).
9. Petit, F. & Furstoss, R. Microbiological transformations. 25. Enantioselective baeyer-villiger oxidation as a tool for the synthesis of enantiopure bicyclic furofuran and pyrofuran chirons. *Tetrahedron: Asymmetry* **4**, 1341–1352 (1993).
